# Machine Learning Demonstrates Dominance of Physical Characteristics over Particle Composition in Coal Dust Toxicity

**DOI:** 10.1021/acs.est.3c08732

**Published:** 2024-01-08

**Authors:** Conchita Kamanzi, Megan Becker, Johanna Von Holdt, Nai-Jen Hsu, Petr Konečný, Jennifer Broadhurst, Muazzam Jacobs

**Affiliations:** † Department of Chemical Engineering, Minerals to Metals Initiative, University of Cape Town, Cape Town 7701, South Africa; ‡ Department of Chemical Engineering, Centre for Minerals Research, University of Cape Town, Cape Town 7701, South Africa; § Department of Environmental and Geographical Science, University of Cape Town, Cape Town 7701, South Africa; ∥ Welcome Centre for Infectious Diseases Research in Africa, Institute for Infectious Diseases and Molecular Medicine, Division of Immunology, Department of Pathology, University of Cape Town, Cape Town 7935, South Africa; ⊥ Neuroscience Institute, University of Cape Town, Cape Town 7935, South Africa; # National Health Laboratory Service, Johannesburg 2193, South Africa

**Keywords:** partial least squares regression, coal mine dust, cytotoxicity, oxidative stress, particle-cell relationships

## Abstract

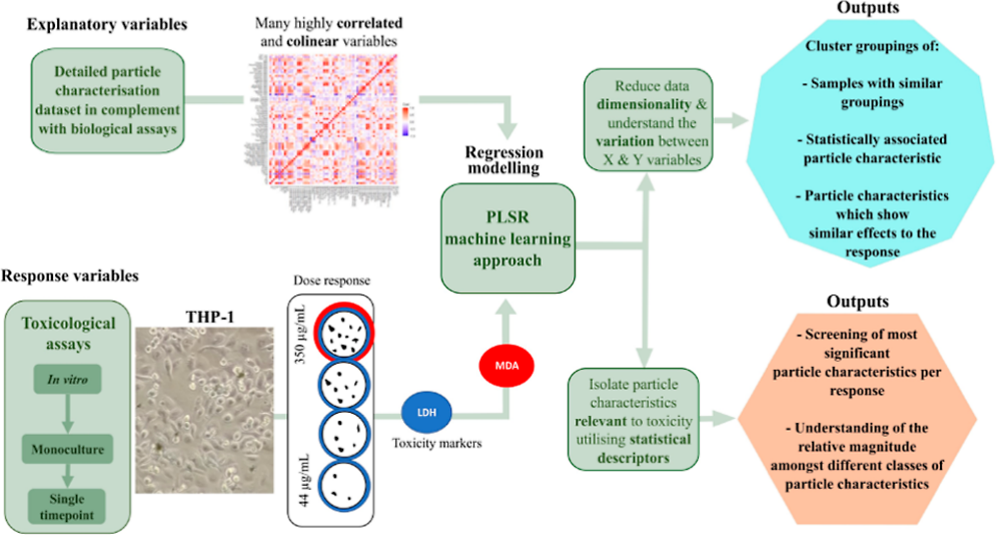

Mine dust has been linked to the development of pneumoconiotic diseases such as silicosis and coal workers’ pneumoconiosis. Currently, it is understood that the physicochemical and mineralogical characteristics drive the toxic nature of dust particles; however, it remains unclear which parameter(s) account for the differential toxicity of coal dust. This study aims to address this issue by demonstrating the use of the partial least squares regression (PLSR) machine learning approach to compare the influence of D_50_ sub 10 μm coal particle characteristics against markers of cellular damage. The resulting analysis of 72 particle characteristics against cytotoxicity and lipid peroxidation reflects the power of PLSR as a tool to elucidate complex particle-cell relationships. By comparing the relative influence of each characteristic within the model, the results reflect that physical characteristics such as shape and particle roughness may have a greater impact on cytotoxicity and lipid peroxidation than composition-based parameters. These results present the first multivariate assessment of a broad-spectrum data set of coal dust characteristics using latent structures to assess the relative influence of particle characteristics on cellular damage.

## Abstract 2

Our machine learning model reviled the influence of dust morphology on macrophage internalization and the subsequent impact of phagocytosis on the potential duration and extent of coal dust biogeochemical reactivity.

## 1 Introduction

Mine dust remains a critical issue for the respiratory health of both workers in occupational settings and communities proximal to mines.^1−4^ Historically, mine dust has been linked to the development of pneumoconiotic diseases such as silicosis and coal workers’ pneumoconiosis (CWP). Epidemiological research has shown that CWP remains a pertinent occupational dust disease, accounting for 25% of global pneumoconiosis cases.^5^ Additionally, chronic respiratory diseases such as bronchitis and emphysema are also known to result from coal dust exposure.^6^

By investigating the pathways leading to coal mine dust pathology, several mechanistic studies have attributed cell and tissue damage to perpetual cycles of stress and inflammation.^7−11^ These cycles are sustained by a feedback of biogeochemical interactions between deposited particles and lung physiology, producing both cell and particle-mediated reactive oxygen species (ROS). Under these circumstances, perpetuated oxidative damage leads to the synthesis and secretion of inflammatory mediators, in addition to the production of growth factors and lysosomal enzymes.^12,13^

In assessing the role of particle characteristics in the molecular and cellular mechanisms leading to coal mine dust toxicity, characteristics such as particle surface area and reactivity, shape, bioavailable iron, and free crystalline quartz content have been identified as agents responsible for cellular damage in isolation.^14−19^ Specifically, these characteristics have been found to contribute to ROS production, lipid peroxidation, cytotoxicity, and collagen production.^12,14,20−24^ While these studies have demonstrated the importance of individual particle-based parameters in the generation of ROS and proinflammatory responses, the complex relationships between the characteristics of coal mine dust and their combined effect on pulmonary cells remain unclear.

To address the lack of clarity, multiple linear regression analysis has been applied to a large array of particle parameters to understand their relations to markers of cellular stress.^25−27^ By regressing pairwise combinations of geochemical and mineralogical parameters on the measures of oxidative potential, several statistically significant relationships explaining the variability in the response could be interpreted. However, the application calls for the construction of a multitude of parameter combinations that would then need to be filtered based on statistical significance. Furthermore, the analysis of any coefficients from these models could potentially be impacted by collinearity, a pertinent issue for large data sets of complementary information (such as geochemical and mineralogical data). Moreover, such an approach does not provide a holistic understanding of how the variation in the sample characteristics impacts the response or how the magnitude of their influence can be compared between different classes of particle characteristics.

Currently, there is no systematic and reproducible application of multivariate analysis to define dependency relationships among a large array of variables in the context of linking coal dust characteristics and cellular responses. This study aims to demonstrate the use of a partial least-squares regression (PLSR) machine learning approach to determine the interrelationships between a large array of physicochemical and mineralogical characteristics of coal particles and markers of cytotoxicity and oxidative stress. In doing so, the study shows the relative significance of an array of physicochemical and mineralogical characteristics to different levels of cytotoxicity and lipid peroxidation among different coals. As part of this application, the study further demonstrates the significance of discriminant analysis as a screening tool for the selection for variables that can represent the “toxic potency” of a set of coal dust samples representative of various stages of the coal washing process. In this context, the study presents a reproducible application of multivariate analysis that can be used to assess the characteristics of dust-sized coal particles which strongly influence cellular damage and stress, while discussing the relevance of different data classes in describing variability in the toxic responses observed. However, when interpreting the study results, it should be understood that the responses considered can only be considered as proxies for human health.

## 2 Methods and Materials

### 2.1 Coal Particle Samples

A set of 17 bituminous coals from collieries based in South Africa, Brazil, the USA, and Mozambique were used to obtain a spectrum of different coal particle populations (Table S1). A detailed description of the sample preparation procedure was given in previous work.^28^ To further understand the distribution of particle sizes in each sample, laser diffraction using a Mastersizer 2000 was employed. Across all the samples, the average size of particles was ∼10.9 ± 1.8 μm (Table S2).

### 2.2 Mineralogical Data

Composition-based data of coal mine dust are conventionally described in the context of mineral/phase identification and element content. X-ray diffraction (XRD) analysis was employed to positively identify minerals based on their crystal structure–refer to Table S3 for a full description of the minerals identified and S1 for additional information on the XRD instrument methodology. The estimation of carbonaceous content was applied using eq 1. This estimate was used to normalize the measured mineral abundances based on the proportion of carbonaceous matter in each sample.
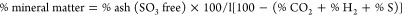
1

In addition to the XRD analysis, the particles were mineralogically mapped by using a FEI quantitative evaluation of materials by scanning electron microscopy (QEMSCAN) 650F auto-SEM-EDS instrument. A further detailed description of the sample preparation and instrument setup in relation to the auto-SEM analysis has been described in previous work.^28^ To assess the accuracy of the mineralogical data defined by QEMSCAN, the data were compared to XRD determined composition (Figure S1).

### 2.3 Chemical Data

An understanding of the major element distributions was established by assaying each coal by means of an X-ray fluorescence (XRF) analysis. Each coal sample was milled, split, and then homogenized into a fusion disk which was subsequently analyzed using a Panalytical Axios Wavelength Dispersive spectrometer, with a full description of the disk preparation and measurement settings in S2. To further understand how these elements are distributed among host minerals, the mineral maps defined by the QEMSCAN were used to quantitatively assess the distribution of the major elements among the mineral groups identified.

### 2.4 General and Mineral Specific Particle Characteristics

In earlier work, coal particle characteristics were grouped as general characteristics (shape and size) and mineral-specific characteristics (mineral liberation), based on the types of data generated from the auto-SEM-EDS characterization of these particles using QEMSCAN.^28^

Of the general characteristics, particle size distribution (PSD) was based on their equivalent circular diameter (ECD). Morphology was quantified from the auto-SEM generated 2D cross-sectional particle maps and based on particle roughness and shape (Figure S2).

For the mineral-specific characteristics, the textural associations between minerals/amorphous phases were defined by the liberation classes (Figure S3).

Additionally, specific surface area (SSA) and crystallite size (CRY) were added to the general and mineral-specific characteristics respectively–S3 and S4 respectively detail the Brunauer–Emmett–Teller (BET) measurement settings and XRD software analysis protocol to obtain the results.

### 2.5 Cell Culture

THP-1 cells (a human leukemia monocytic cell line) purchased from ATCC (American Type Culture Collection) were cultured in T75 tissue culture-treated flasks (Greiner CELLSTAR) with RPMI 1640 culture medium supplemented with 10% fetal calf serum (Gibco) and 1% penicillin–streptomycin (Gibco) at 37 °C in a humidified 5% CO_2_ atm. To assay for the compounds relevant to cytotoxicity and lipid peroxidation, a generalizable experimental setup was developed to assess the toxicity of the 17 coal particulate samples based on prior *in vitro* experimental procedures described.^13,29−31^ For all the assays conducted in this study, cellular exposure was carried out by removing the culturing media 24 h after seeding cells with 50 ng/mL phorbol 12-myristate-13-acetate and replacing it with UV-sterilized media-coal suspensions.

### 2.6 Lactate Dehydrogenase Assay

Cell death via cytosolic damage was measured via a lactate dehydrogenase (LDH) colorimetric assay kit (Roche). Flatbottom 96-well plates were seeded at 2.3 × 10^4^ cells/well (∼40% confluency) in a final volume of 200 μL/well. A particle concentration gradient of 350, 175, 88, and 44 μg/mL was used for the cytotoxicity assay. Each experimental well was replicated only once due to the high volume of samples and limited availability of reagents. In addition to the experimental wells, control assays included blank wells (only media) as well as negative and positive controls (media only and 0.1% Triton X-100 respectively) were added to each plate.

At the end of the 72 h exposure period, the cell death was measured according to the manufacturer’s instructions where 100 μL of the assay solution was added to a 96-well ELISA microplate (Thermo Scientific) and the absorbance read at 490 nm using an ELISA reader (VersaMax, Molecular Devices). The results were presented as a percentage of the positive and negative controls (Triton X-100 and RPMI 1640 only respectively).

### 2.7 Lipid Peroxidation Assay

The extent of ROS related damage via free radical attack of lipid cell membranes was measured using a lipid peroxidation—malondialdehyde (MDA) assay kit (Sigma-Aldrich). Generally, this type of assay is widely used as an indicator of oxidative stress; however, it should be acknowledged that the assay is nonspecific for different aldehydes. 12-well plates were seeded at a density of 1 × 10^6^ cells/well in medium in a final volume 1 mL/well. Based on prior optimization studies conducted with the LDH assay, the exposure conditions for the lipid peroxidation assay were set at a particle concentration of 350 μg/mL—incubated for 72 h–to obtain a strong measurement signal. Notably, the lipid peroxidation assay was only conducted on coals that displayed cytotoxicity greater than 10% at this concentration, due to limitations in reagent availability. After the 72 h exposure period, the sample was prepared and analyzed according to the manufacturer’s instructions. Finally, 200 μL of the reaction solution from each sample was pipetted into a 96-well microplate where the absorbance was read at 532 nm using an ELISA reader (VersaMax, Molecular Devices). The final MDA concentration was quantified by comparing samples to a standard curve determined synchronously with the samples.

### 2.8 Statistical Analysis

To assess the relative significance of the 72 coal particle characteristics (*X* variables) to the resulting exposure-based responses expressed in the 17 samples *in vitro* (*Y* variables), a PLSR machine learning approach was used. Generally, the PLSR operates by regressing the *X* and *Y* variables as a function of the product of two smaller matrices called scores (latent components) and loadings.^32^ In its final form, *Y* is regressed by the *X* scores instead of *X* (in the form of a linear regression *Y* = *BX*). This allows the model to perform in cases where variables are colinear and where there are more variables than samples.^33^ The PLSR model was conducted in R 4.0.3 (RStudio Team, 2020) using the pls package^34^ with the SIMPLS algorithm^35^ on standardized data with a cross-validation (CV) step to assess the model performance per component generated.

Upon initialization, the model was allowed to define as many components needed to maximally explain the variance in both *X* and *Y*, while computing the cross-validated predictions per component (Table S7). From this process, the PLSR determined 3 and 4 components which accounted for 91 and 98% of the variance in the LDH and MDA responses, respectively. As overfitting is a common issue for PLSR due to the number of correlated variables, the cross-validated predictions per component were chosen to assess whether the model was subject to overfitting. In doing so, the difference between the RMSEP (root mean squared error of prediction) generated from the model with n components (CV) and the cross-validated model with the same number of components (adjCV) was computed and considered based on their similarity—(Table S4). Based on the results, both the LDH and MDA models were deemed representative of the sample variation in responses with reasonable explanatory capacity for the *X* variables. This was based on an assessment of the similarity between the RMSEP and adjCV, as well as the model performance determined *R*^2^ of predictions versus observations (see Table S4).

## 3 Results

### 3.1 Advanced and Targeted Particle Characterization of Coal Dust Yields In-Depth Understanding of Intersample Variability

#### 3.1.1 Mineralogical and Elemental Distributions Using Auto-SEM Analysis Provide Mapped Linkages between Elements and Their Host Minerals

**Figure 1:**
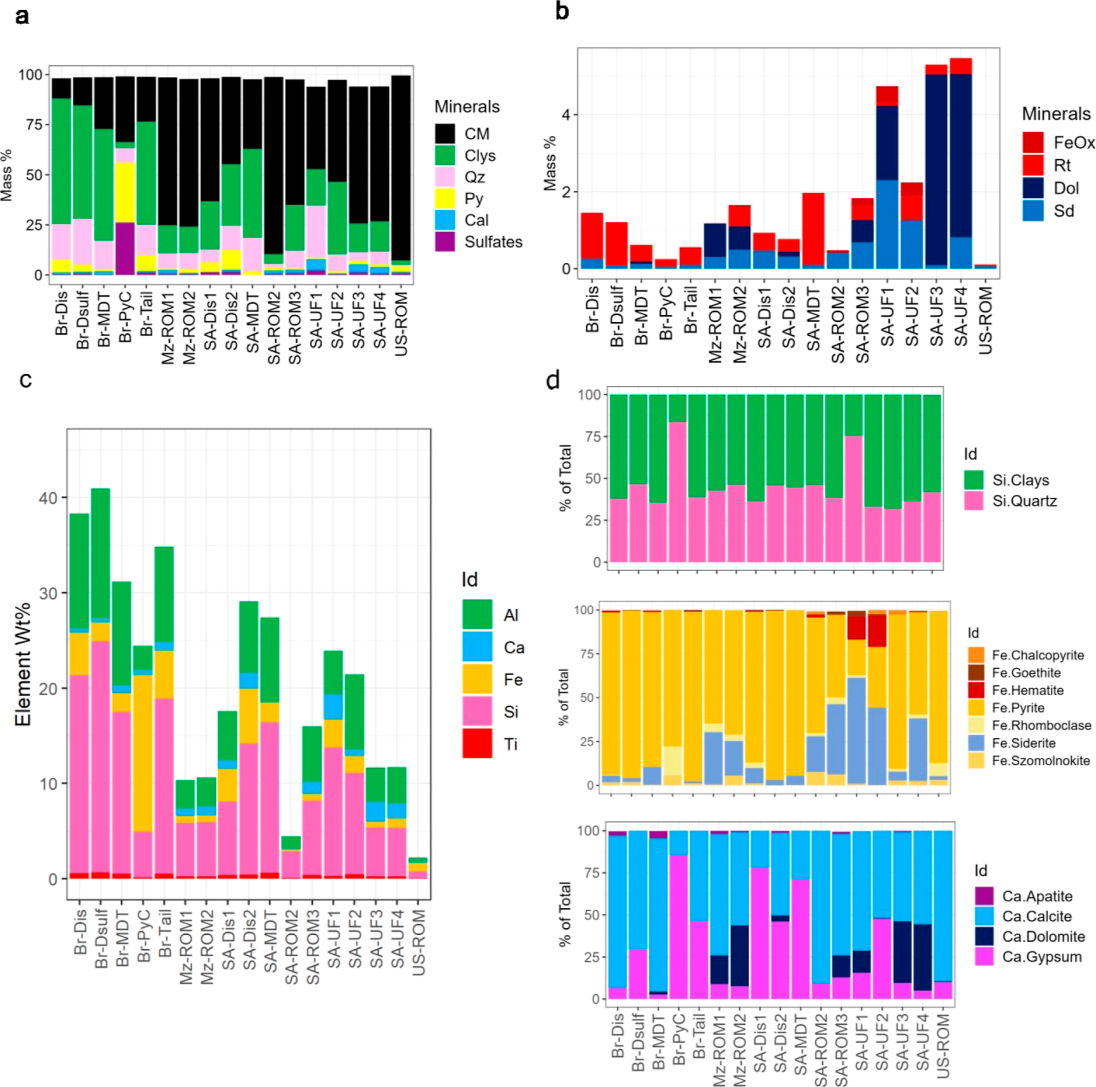
Summary of the major and minor compositional characteristics of the coal dust particles as determined by QEMSCAN analysis. Major mineral constituents were identified where CM = carbonaceous matter, Clys = clays, Qz = quartz (SiO2), Py = pyrite (FeS2), Cal = calcite (CaCO_3_). In the context of the clays and sulfates, these categories comprise kaolinite [Al_2_Si_2_O_5_(OH)_4_] and Illite [K_0.6_(H_3_O)_0.4_Al_1.3_Mg_0.3_Fe_0.1_^2+^Si_3.5_O_10_(OH)_2_·(H_2_O)], gypsum [Ca(SO_4_)·2(H_2_O)], szomolnokite [Fe^2+^(SO_4_)·(H_2_O)], jarosite [KFe^3+^(SO_4_)_2_(OH)_6_], and rhomboclase [HFe^3+^(SO_4_)_2_·4(H_2_O)] respectively. (b) Minor mineral distribution among samples, where FeOx, Rt, Dol, and Sd refer to iron oxide minerals, rutile (TiO_2_), dolomite [CaMg(CO_3_)_2_] and siderite [Fe^2+^(CO_3_)] respectively. For reference, the minerals classed as iron oxides were hematite (Fe_2_O_3_) and goethite [Fe^3+^O(OH)]. (c) Major element concentrations measured using XRF spectrometry and calculated as the wt % element analyzed. Carbon and oxygen represent the remainder as loss on ignition. (d) Distribution of the elements Si, Fe, and Ca, among the mineral hosts identified. The data are represented as a percentage relative to the total amount of Si, Fe or Ca shown in Figure 1c.

Automated scanning electron microscopy (auto-SEM) was used to quantify the sample composition alongside the distribution of elements among their mineral hosts. From the resulting analysis, over 90 wt % of the sample mass comprised carbonaceous matter, clays, quartz, pyrite, sulfates, and calcite (Figure 1a and Table S5 for mineral formulas). Trace mineral phases such as siderite, dolomite, rutile, and iron oxides contributed <5.5 wt % of the sample mass (Figure 1b). The measured major element concentrations (Al, Ca, Fe, Si, and Ti)—Figure 1c, showed a broad variation between the samples (43 and 2 wt %). When normalized, Si and Al constituted most of the element content (76 ± 15%) with Ca, Fe, and Ti contributing to the balance (21 ± 15%).

Si was mainly distributed between quartz (54%) and clays (45%)—Figure 1d. Generally, most of the Fe in the samples was hosted in pyrite (∼74%) with appreciable amounts of Fe distributed in carbonate (siderite), sulfate (rhomboclase and szomolnokite) (∼23%), and minor abundances of iron oxide sources (hematite and goethite −0.8 and 3.4%, respectively). Ca was also hosted in multiple minerals, including carbonates (calcite −61% and dolomite −15%), sulfate (gypsum −29%), and phosphate-bearing minerals (apatite −0.2%).

Ultimately, the results show that the elemental distribution between host minerals is complex and variable among different coal samples.

#### 3.1.2 Particle Size, SSA, and Compositional Factors of Crushed Coal Do Not Fully Account for Fine Dust Generation

The PSD was measured to understand the proportion of particles reporting to different size categories based on their ECD–details on the PSD across size fractions are given in Table S2. The finer size fractions (<5 μm) showed substantial variations in the proportion of particles (3–30 mass %), indicating that despite experiencing the same crushing treatment, certain coals have a greater propensity to produce fines.

A multilinear regression was used to assess the influence of carbonaceous matter and clay content on the proportion of fines generated. Overall, the abundance of carbonaceous matter and clays in the samples poorly explains the proportion of fines generated by each coal (*R*^2^ = 0.32, *p*-value = 0.0687)—Table S6. This suggests that the amount of clays and carbonaceous matter in coal play a very limited role in the final proportion of fines.

From the PSD data, it was assumed that samples with a higher proportion of fines would yield a larger SSA. However, no relationship was found between the SSA and the percentage of fines. To investigate additional descriptors for the variation in SSA, a regression between SSA and the mineral matter content per samples revealed that SSA and the total mineral content possess a moderate but statistically significant linear relationship (*R*^2^ = 0.55, *p*-value = 0.0007)—Figure S4. This relationship suggests that intrinsic mineral-related artifacts could have an impact on the effective SSA of the sample.

#### 3.1.3 Distribution of Particle Shape and Roughness by Auto-SEM Analysis Shows Morphology Dominance in Crushed Coal Particulates

To obtain an understanding of the morphology of particles, parameters relating to particle roughness and shape were quantified from the auto-SEM generated 2D cross-sectional particle maps. Across the range of coals, most particles tended to be jagged in roughness (35–84% by abundance)—Figure S2a and equant and angular in shape (55 ± 10 and 28 ± 10% respectively)—Figure S2b. These observed biases in the general roughness and shape were accounted for by the action of crushing and milling. By relating the suggested effect of mechanical size reduction to the SSA, PSD, and morphology of the material, we strongly point to the integral role of mechanical breakage as a determinant of several physical characteristics relevant for biogeochemical reactivity.

#### 3.1.4 Analysis of Mineral CRY Shows Intercountry and Coalfield Variation in Quartz, Pyrite and Kaolinite

CRY serves as an indicative parameter for the surface-related reactivity of minerals. Among the dominant minerals (quartz, kaolinite, and pyrite), the kaolinite generally consisted of smaller crystallites (26.8 ± 4.8 nm), in comparison to pyrite (68.3 ± 11 nm) and quartz (69.9 ± 25.9 nm)—Figure S5. This observation is consistent with the fine-grained nature of clays and further suggests that kaolinite particles may have fewer surface defects compared to pyrite and quartz. When the samples were grouped based on the country of origin, the CRY of pyrite was roughly consistent across the countries of origin (mean CRY for BR—71.4 ± 6.8 nm, SA—70.8 ± 10.4 nm, and US—68.3 nm), except for the Mozambique samples (52.1 ± 8.3 nm). For quartz, a wide range in CRY was reported across the different countries of origin. The quartz analyzed from Brazilian coal reported the largest CRY (98.1 ± 7.7 nm). The quartz from South African samples, however, showed a wide distribution of CRY (58.2 ± 24 nm). This indicates that different coalfields may possess quartz grains with different CRY.

#### 3.1.5 Mineral Liberation and Association Show Crushed Coal Particulates Mainly Occur as Composites

Using the parameters mineral liberation and mineral association, the textural relationships between the biologically relevant minerals (quartz, clays, and pyrite) were assessed to provide additional information about the potential reactivity of these minerals in the biological context. In terms of texture, all three minerals generally occurred as composite particles associated with other minerals rather than liberated grains—Figure S6. For quartz, 17 ± 11 and 28 ± 9% were fully and mostly encapsulated (unliberated), respectively, while 32 ± 17% were fully and 23 ± 6% moderately liberated. In the case of clays, 9 ± 10% were fully and 25 ± 11% mostly encapsulated, respectively, while 5 ± 20% were liberated, and a further 32 ± 8% moderately liberated. For pyrite, 15 ± 14% of the particles were reported as liberated and 33 ± 22% were reported as moderately liberated. On the opposing end of the spectrum, 15 ± 11 and 33 ± 22% of the pyrite particles were fully and mostly encapsulated, respectively.

### 3.2 Dust-Sized Coal Particulates Show Differential Cytotoxicity and ROS-Related Damage in Human Macrophages

**Figure 2:**
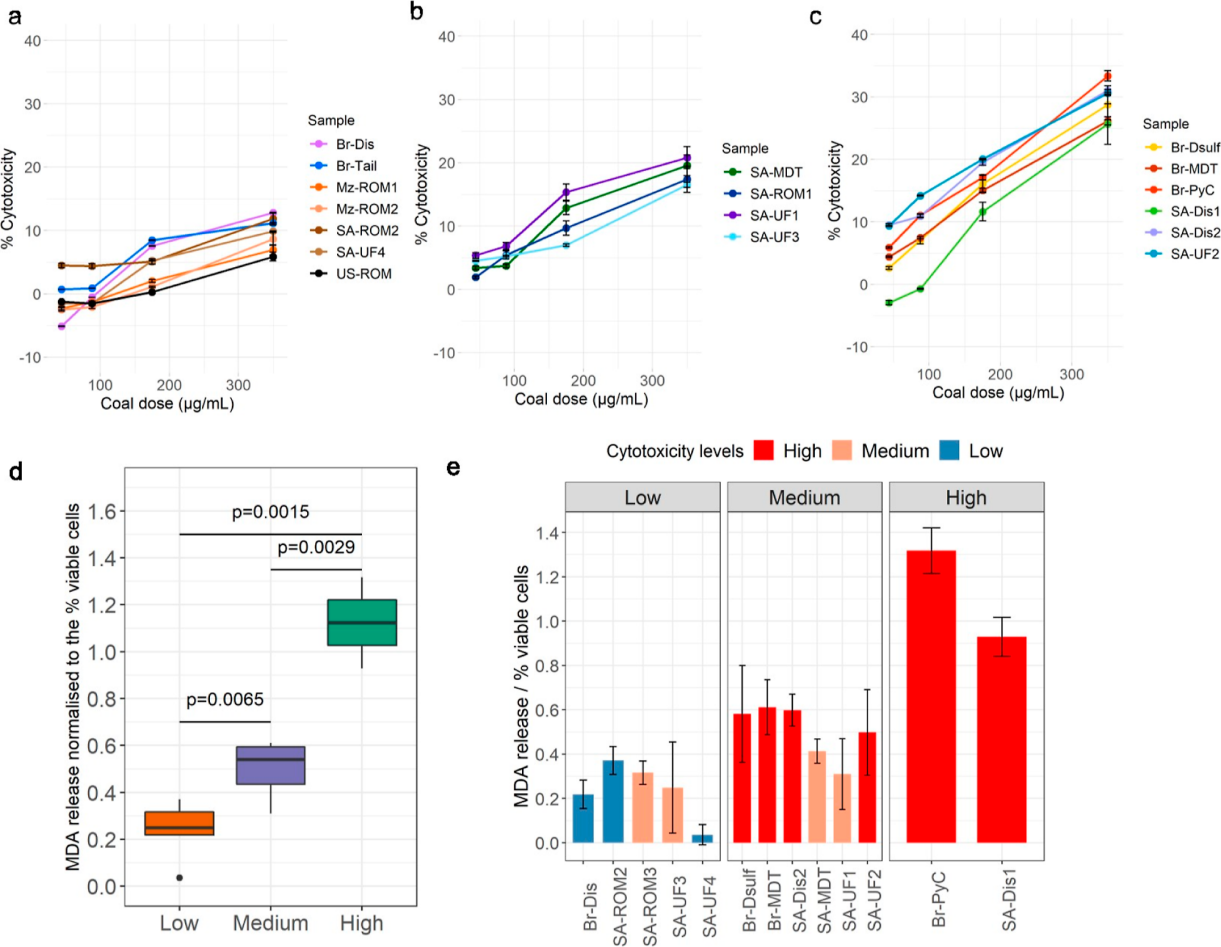
Differential expression of cytotoxicity and lipid peroxidation among coal dust exposed THP-1 cells (72 h time point). (a–c) represent the samples that have been subdivided into high, medium, and low cytotoxic responses, respectively, based on clusters observed at the 350 μg/mL concentration and the gradient of samples in these clusters. For reference, the relative error reported at each concentration was less than 3.5% across the samples analyzed. (d) displays the distribution of MDA produced by the THP-1 cells from a 72 h exposure with 350 μg/mL of the particle samples. Based on the results three groups of samples could be identified, namely low, medium, and high MDA-release samples. (e) further classes the samples based on their measured cytotoxic response. The amount of MDA released relative to the negative control was normalized by the percentage of viable cells based on the results of the cytotoxicity assay. For reference, the relative error across all measurements was less than 0.2 units.

Cytotoxicity was assessed across a series of dust doses, where three distinct classes of samples were identified based on their trends. These classes corresponded to samples that reported either low (L), moderate (M), or high (H) cytotoxicity (Figure 2a–c respectively). The dose–response relationships were modeled using a linear regression to determine whether the gradient of each sample could be used as a summary descriptor for the intensity of cytotoxic response (results represented in Table S7). Additionally, the average gradient of samples in each cytotoxicity class was compared via a *t*-test to verify the significance of the classes. The results confirmed that the differences between samples in the cytotoxicity classes can be attributed to the differential rate of cytotoxicity as a function of dose [*p*-value (M–L) = 0.03; *p*-value (H–L) = 0.0007; *p*-value (M–H) = 0.00001]. At the highest dust concentration (350 μg/mL), the samples in classes L, M, and H ranged from 5.87 ± 0.69 to 12.80 ± 0.07, 16.52 ± 0.41 to 20.82 ± 1.73, and 25.66 ± 3.24 to 33.36 ± 0.83% cytotoxicity, respectively. These results demonstrated that the cytotoxicity of the different coal particles can be explained by a similar mechanism (which can be modeled linearly), although the intensity of the response may differ due to factors other than dose.

To assess the extent of radical-related damage caused by the coal particle-cell interactions, the expression of MDA–a chemical biproduct of lipid peroxidation reactions–was measured. The results were sorted and grouped into three statistically distinct classes reflecting low, medium, and high response samples (Figure 2d). To understand whether the cytotoxicity classes overlap with those of lipid peroxidation, the level of MDA release per sample was mapped with the corresponding cytotoxicity level (Figure 2e).

### 3.3 PLSR Latent Structures Demonstrate an Effective Tool for Identifying Particle-Cell Relationships

**Figure 3:**
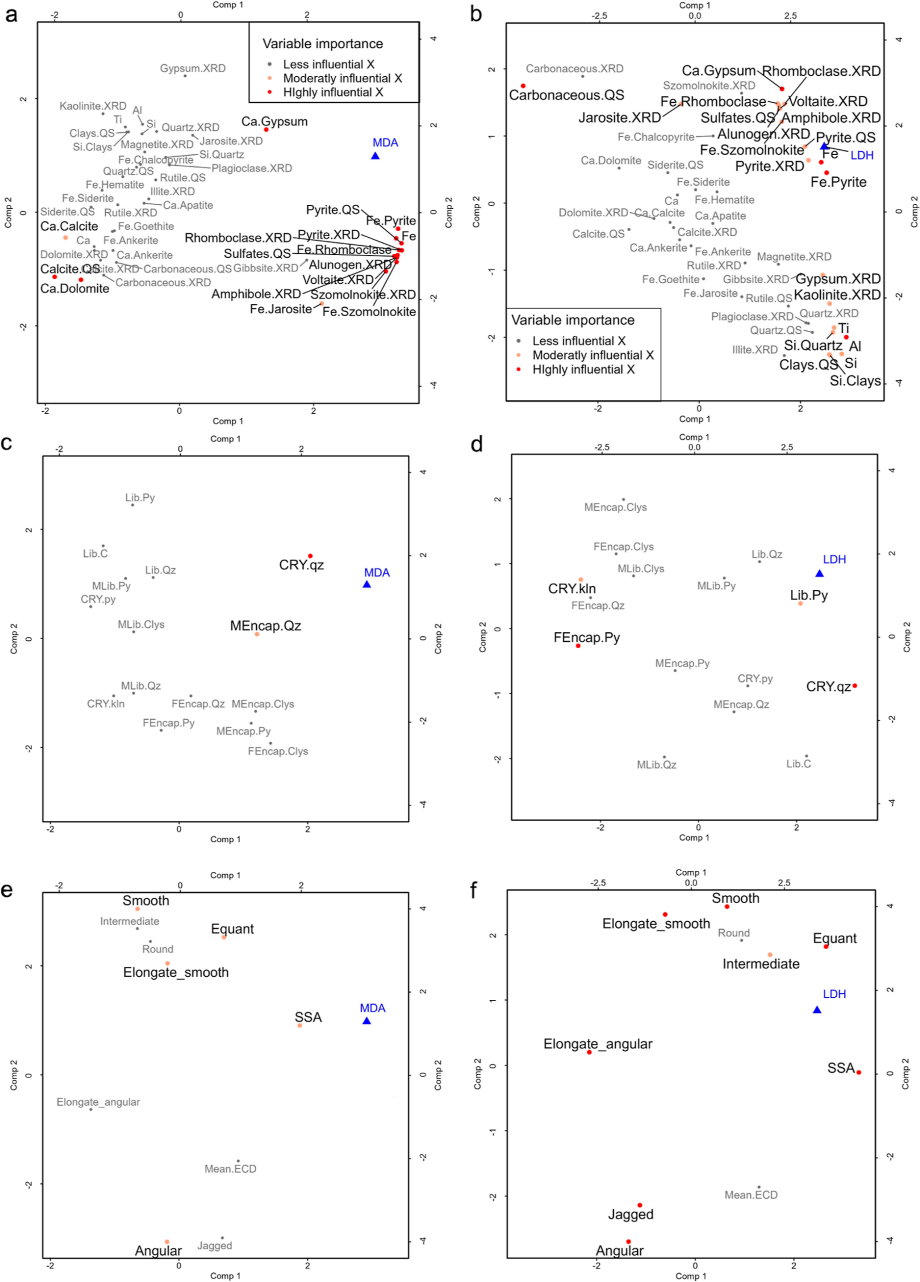
Loading plots representing the particle parameters that are either positively or negatively correlated, as well as the parameters correlated with MDA or LDH releases. To interpret the loading plot, parameters that cluster together display an association suggesting they behave similarly, while parameters that oppose each other show opposing effects in the model system. The particle parameters are represented by the red, orange, and gray dots, and the two responses are indicated by blue triangles. (a,b, c,d and e,f) represent pairs of loading plots categorized by mineral and element-based data, mineral-specific parameters, and general particle characteristics, respectively. The pairs contrast the two responses where (a,c,d) related to the MDA-based model and (b,e,f) relate to the LDH-based model. For (a,b) the minerals have the XRD and QS, this indicates that the value was derived from XRD and QEMSCAN analysis, respectively. For C and D the liberation state and CRY data for the minerals quartz = qz, pyrite = py, and clays = clys (kaolinite = kln) are abbreviated as follows: Lib = liberation, MLib = moderately liberated, MEcap = mostly encapsulated, FEncap = fully encapsulated, and CRY = crystallite size. VIP scores were used to class the characteristics by importance to the model, where the influence of each variable was categorized as highly (VIP > 1.25), moderately (1 < VIP < 1.25), or less influential (VIP < 1). The total variance explained by components 1 and 2 across all variables in the MDA model were comp1 = 21.86%, comp2 = 15.09%. The total variance explained by components 1 and 2 across all variables in the LDH model comp1 = 19.94%, comp2 = 12.39%.

Loading plots were used to understand correlated parameters and the contribution of each particle characteristic (*X* variable) and response (*Y* variable) to the first two components of each response model. To aid in the interpretability of the plots, the characteristics were broken into groups representing: mineral and element-related data (Figure 3a,b); mineral-specific-data–libation and CRY—(Figure 3c,d); and general particle characteristics–physical characteristics—(Figure 3e,f). VIP scores were used to distinguish the relative significance of each characteristic to the model. The proximity and direction (from the origin to each plotted variable) among parameters were considered to assess whether parameters are correlated (in proximity) and either positively (vectors of the same direction) or negatively (vectors of opposing directions) associated.

For lipid peroxidation, the Fe-bearing sulfate and sulfide minerals are positive and closely associated with MDA release (Figure 3a). Similarly, for cytotoxicity, the Fe-bearing sulfate and sulfide minerals were both positively associated with cytotoxicity–measured by the release of LDH, although Fe-sulfate minerals had a more moderate influence on the release of LDH than on MDA (Figure 3b).

Gypsum hosted Ca additionally showed a positive association to MDA release; however, calcite and dolomite hosted Ca displayed a negative association to the response. Ca distributed in gypsum and the total Ti content were also defined as influential and positively associated parameters to LDH release (Figure 3b), while Si and Al, and their main host minerals–quartz and kaolinite respectively–both showed a moderately influential and positive association. Carbonaceous content was found to be highly influential and negatively associated with cytotoxicity. This suggests that the composition-based effects related to cytotoxicity are a function of the mineral matter and not carbonaceous content.

In terms of mineral specific characteristics, the CRY of quartz (CRY.qz) was found to be positively associated and highly influential on both MDA (Figure 3c) and LDH release (Figure 3d). The CRY of kaolinite (CRY.kln) displayed a moderate influence on the model with a negative association with LDH release (Figure 3d). Mostly encapsulated quartz displayed a moderate level of influence to the MDA-based model. For the LDH-based model, liberated pyrite grains showed a moderate positive association to cytotoxicity, while fully encapsulated pyrite displayed a highly influential negative association to the response.

For the general particle characteristics, the SSA shows a positive and close association to both MDA (Figure 3e) and LDH release (Figure 3f). Particle shape had a moderate and strong influence on MDA and LDH release respectively, with equant-shaped particles having a positive effect, and particles classified as “angular” and “elongate_smooth” having a negative effect. Based on the near orthogonal trajectory of the particle shape and SSA vectors, this suggests that the effect of these parameters on LDH release could occur via different mechanisms. Roughness additionally has a strong influence on LDH release, with the opposing direction of the smooth and jagged particles, suggesting that these properties share a similar magnitude of influence but have opposing effects (Figure 3f).

### 3.4 PLSR Coefficient Analysis Demonstrates a Screening Tool for Assessing Relative Significance of Coal Dust Characteristics to Cytotoxicity and Oxidative Stress

To assess the contribution of each variable to the MDA and LDH-based models, coefficient plots were constructed which further utilized the VIP scores of each variable to screen for the characteristics that significantly influenced the respective responses of each model.

**Figure 4:**
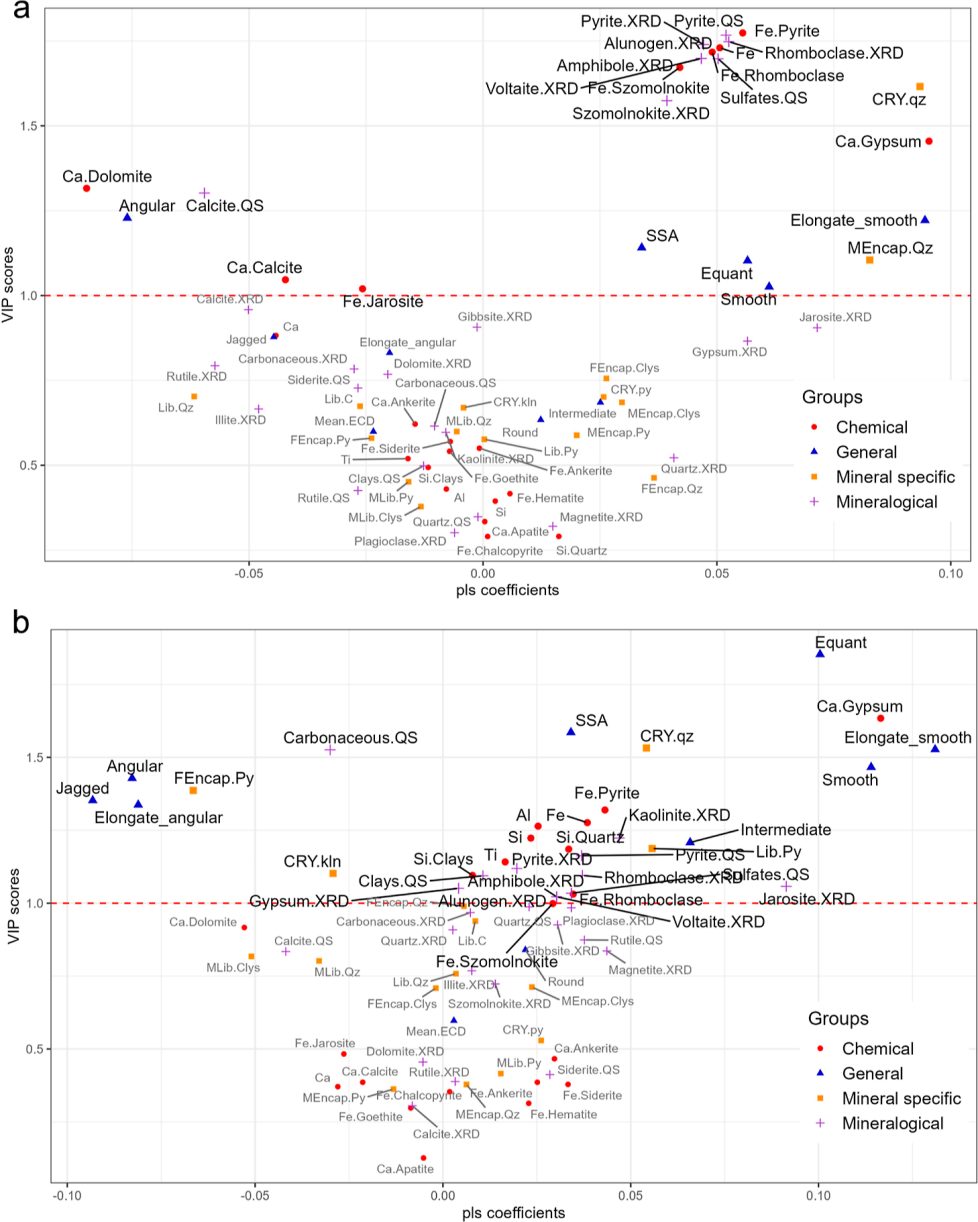
Coefficient plot comparing the relative impact of the particle characteristics based on their coefficient value against the measure of relevance computed for each parameter in relation to the model (based on VIP scores). A and B represent the coefficients plots for the MDA and LDH-based models, respectively. To interpret the coefficients plot, the *x*-axis represents the model coefficient in the form *Y* = *BX*, where *B* is the coefficient and is calculated for each explanatory variable “*X*” included. The magnitude of the coefficient gives an indication of the relative importance of the parameter, and the direction (positive or negative) yields whether the parameter either promotes or depresses the response. The *Y*-axis represents the VIP score calculated for each parameter. This stratifies which parameters played a significant role in the model. A VIP score >1 was used as the threshold for significance.

Of the significant characteristics in the MDA-based model (VIP > 1), CRY of quartz, the presence of elongate and smooth particles, and the amount of Ca hosted in gypsum have the highest positive coefficient values compared to other parameters (Figure 4a). Although the encapsulated quartz reported a coefficient value similarly high to these parameters, the raw data indicated that the level of uncertainty between coals displaying different levels of MDA release was insignificant.

Other characteristics showing significant positive coefficient values include the abundance and distribution of Fe in Fe-bearing sulfate and sulfide minerals, as well as particles that are smooth and equant. SSA was found to promote MDA release to a lesser extent compared to the CRY of quartz and composition-based parameters. By contrast, the abundance and distribution of Ca in carbonate minerals, along with angular-shaped particles, displayed large negative coefficient values. Fe distributed in jarosite displayed a moderately negative coefficient value.

Of the characteristics significant to the LDH-based model, particle shape showed a strong influence on cytotoxicity compared to composition-based parameters (Figure 4b). Specifically, equant particles with smooth roughness, as well as elongate and smooth-shaped particles, displayed a large positive coefficient value, while more jagged and angular particles showed a large negative coefficient value. Particles with intermediate roughness displayed a moderate but positive coefficient, indicating that such particles have less influence on LDH release than equant and smooth particles. Gypsum hosted Ca was the only composition-based characteristic that displayed a large positive coefficient–of similar magnitude to that of equant and smooth shaped parameters.

Mineral-specific characteristics, such as pyrite liberation and the CRY of quartz and kaolinite, had a moderate impact on the promotion of cytotoxicity. The coefficient values for pyrite liberation show that fully encapsulated pyrite particles have a relatively large but negative coefficient value, whereas liberated pyrite has a moderately positive value. This suggests that the negative effect of fully encapsulated pyrite has a stronger influence on LDH release compared to the positive effect of liberated pyrite. The CRY of quartz displayed a moderate positive coefficient compared to that of kaolinite–which showed a moderate negative value. The coefficient value of the carbonaceous content was observed to be similar to that of kaolinite CRY, which suggests that more amorphous structures may have a negative effect on LDH release.

Across other composition-based characteristics, the abundance of jarosite was observed to have the highest positive coefficient value among the minerals that promote cytotoxicity. Fe distributed in pyrite as well as other Fe-bearing sulfates and kaolinite abundance were observed to cluster with moderate coefficient values. Si distributed in quartz displayed a moderate but positive contribution to LDH release, despite the VIP score <1 for quartz abundance. Similarly, Ti content was observed to have a moderate but positive contribution to the model even though the main Ti-bearing phase rutile reported a VIP score <1. Lastly, SSA displayed a moderate but positive contribution to cytotoxicity, which yielded a coefficient value in a range similar to the mineral and element data. This shows that particle SSA has a similar impact on LDH release as composition-based characteristics.

## 4 Discussion

One of the greatest challenges faced by studies that seek to relate the characteristics of coal mine dust to cellular damage is the phenomenon of samples with similar characteristics presenting different levels of toxicity or inflammation. This makes developing a generic understanding of coal dust toxicity from single or small groups of parameters (such as pyrite or quartz content) infeasible.

The results presented show not only that coal particulates from different parental sources display differential cytotoxicity and lipid peroxidation but also that samples which displayed the highest levels of cytotoxicity generally elicited a higher release of MDA than samples which showed low cytotoxicity. Such findings are consistent with correlative links between lipid peroxidation, markers of oxidative stress, and cytotoxicity.^19,36−39^ This may further imply that the mechanisms related to oxidative stress may also influence the level of cytotoxicity.^14,40^

Apart from the differential toxicity of coal dust, the relative significance of different physicochemical and mineralogical characteristics of coal dust in relation to their impact on the known pathogenic pathways leading to diseases such as CWP is still poorly understood. By using PLSR loadings and coefficient plots, coupled with added refinement of the VIP scores, this study was able to demonstrate a systematic screening approach to determine the most significant characteristics for toxic responses on a cellular level. Additionally, the study presented the first comparative analysis of the effect of 72 physicochemical and mineralogical characteristics on both oxidative stress and cytotoxicity expressed from exposed macrophages.

Between the different classes of characteristics (mineral chemistry and composition, mineral-specific properties such as CRY and liberation, and general particle characteristics including surface area, shape, and roughness), particle shape and roughness were observed to have the highest influence on both cytotoxicity and lipid peroxidation. More particularly, equant and smoother shaped particles were found to promote cytotoxicity in macrophages, whereas more angular and jagged particles displayed the inverse effect. Such results are congruent with reports that equant particles with a block-like/ball-like habit tend to promote incomplete phagocytosis compared to angular and jagged particles which promote complete phagocytosis.^16,41^ In the former case, it is expected that macrophages which are unable to successfully internalize their target undergo a state of “frustrated phagocytosis” which promotes the generation of ROS and inflammatory indicators.^17,42^ Considering that particle shape was given a higher priority than compositional based parameters in both models, it is possible that the unsuccessful internalization of particles allows for a longer period of biogeochemical reactivity in the extracellular environment compared to completely internalized particles. By extension, an extended period of biogeochemical reactivity involving deleterious composition-based characteristics could trigger both direct and indirect cell damage.^39^

Regarding mineral chemistry, Fe associated with the sulfide mineral pyrite and Fe-sulfate minerals rhomboclase (HFe(SO_4_)_2_·4H_2_O) and szomolnokite (FeSO_4_·H_2_O) played an influential and similar role in both lipid peroxidation and cytotoxicity. This observation suggests that both pyrite and its soluble iron sulfate alteration products ( formed in acidic environments) serve as a source of bioavailable iron, which can aggravate the levels of Fenton-based reactive oxygen species (ROS), leading to lipid peroxidation and eventual cytotoxicity. The negligible to negative influence of the iron sulfate mineral jarosite [KFe_3_(SO_4_)_2_(OH)_6_] on lipid peroxidation, on the other hand, indicates that some Fe-bearing sulfates may be more reactive than others and thus have different impacts on Fe-mediated radical production.

Gypsum (CaSO_4_·2H_2_O) hosted Ca was also highly influential in both lipid peroxidation and cytotoxicity. The observed relationship between Ca in gypsum and these responses is congruent with the readily soluble nature of gypsum in biological environments, as well as studies which have shown that elevated levels of extracellular and intracellular Ca can disrupt cellular functions which utilize Ca via signaling mechanisms, as well as induce apoptosis.^43,44^ To highlight the potential significance of Ca for future investigation, studies have shown that elevated levels of calcium could lead to the activation and transcription of NF-κB and the nuclear factor of activated T cells in the *in vivo* context, both involved in inflammatory pathways and could further promote cellular stress.^45,46^ Further discussion on the effect of gypsum-hosted Ca on cellular damage is presented in Supporting Information Section 5. Conversely, Ca derived from calcite and dolomite was found to have a strong negative impact on lipid peroxidation. This is most likely due to the buffering capacity of carbonate minerals which alter pyrite to its chemically nonreactive iron hydroxide products.^9^

The CRY of quartz, as opposed to quartz abundance, was determined to be highly influential in lipid peroxidation and less so to cytotoxicity. This suggests that the cytotoxicity of quartz is potentially better reflected by its CRY than its abundance. Mechanistically this could be explained by the positive link between mineral CRY and surface defects.^47,48^ As surface defects are known to serve as sites for ROS generation in biological contexts, the relatively large CRY of quartz compared with other minerals such as kaolinite supports this observation. Although mostly encapsulated quartz displayed a moderate influence on MDA release, this is contradictory to the known surface-based reactivity of quartz. Through an uncertainty analysis between values of samples grouped with this trend, no significant differences were found between the proportion of mostly encapsulated quartz and the level of MDA release (Table S8). Conventionally, the kaolinite content is generally considered to play only a mitigative role in suppressing the toxicity of quartz. However, the LDH-based model in this study defined the effects of kaolinite in coal dust to have a magnitude of influence on cytotoxicity as pyrite. Such findings are consistent with studies which demonstrated a link between kaolinite and cytotoxicity in macrophages.^49−51^ Despite this apparent link, little research has been conducted to further understand the mechanism of kaolinite toxicity.

While previous studies have shown a positive association between Ti content and oxidative potential based on noncellular chemical assays, the results presented in this study indicated that neither Ti content nor its mineral host rutile (TiO_2_) report a significant effect on lipid peroxidation in macrophages.^22,25^ Conversely, the results of the LDH-based model show that the Ti content is moderately influential on cytotoxicity, despite rutile displaying no significant contribution to the model. Separate analysis presented in this study has shown that Ti is highly associated with kaolinite (Figure S7), a finding supported by a study which described the occurrence of TiO_2_-kaolinite aggregates cemented to kaolinite particles in clay deposits.^52^ It is thus possible that the relative significance of the Ti content to cytotoxicity could be a result of its association with kaolinite. As the effects of these aggregates on cellular function have not been discussed in literature, this could be a potential avenue for studies seeking to further understand the mechanics associated with kaolinite-induced cytotoxicity.

Ultimately, the analysis showed that the use of loading plots combined with screening criteria such as VIP scores provides important insights into understanding how different coal dust characteristics relate to one another and markers of toxicity. By assessing the directionality and clustering of parameters, it becomes possible to hypothesize potential concurrent mechanisms or to link the observed associations to already established pathways. These results present the first multivariate assessment of a broad-spectrum data set of coal dust characteristics using latent structures to assess the relative influence of particle characteristics on macrophage toxicity and oxidative damage.

Through the results presented, the application of PLSR was demonstrated to provide a robust understanding of how the characteristics of dust-sized coal particulates influence cellular damage in macrophages, while accounting for collinearity. Ultimately, the application of this protocol to 17 different dust-sized coal samples demonstrated the key differences between samples and their influence on levels of cytotoxicity and lipid peroxidation, which until this study have not been demonstrated by a single regression. Based on the results, it is proposed that while the toxic potency of coal dust is primarily a function of the reactive mineralogical and chemical components within the particles, the impact of the deleterious components is only realized if (1) the dust is allowed to react in the extra-cellular environment, which is highly governed by the shape of the particles, and (2) if the mitigative factors which can either neutralize or suppress the anticipated reactivity are minimal. Although it is acknowledged that the dust particles generated within this study might not be representative of real-world mine dust samples or samples within what is considered the respirable fraction (<4 μm), the model applied here could be applied more broadly to include respirable coal mine dust from site and other types of mine dust in general.

As an overall outcome of the results, this study provides a robust analysis strategy for elucidating particle-cell relations, which can further advance the understanding of coal dust-induced disease pathology. In the broader sense, the model additionally allows for the analysis of multiple response variables that could be impactful in terms of linking mechanistic responses across indicators of cellular stress, inflammation, and cytotoxicity. This information provides a means to understand how certain particle properties could mitigate the toxic effects caused by compositional reactivity and the disruption of phagocytosis. Such strategies would complement recent research assessing novel treatments for coal mine dust-related diseases.^53−55^

## Supporting Information Available

(https://doi.org/10.25375/uct.22309492.v1);immunological responses of THP-1 cells exposed to coal dust *in vitro* (https://doi.org/10.25375/uct.22309357.v1); characterization of dust-sized coal particulates: full physicochemical, mineralogical, and textural data set extracted from QEMSCAN analysis (https://doi.org/10.25375/uct.22309117.v1); characterization of dust-sized coal particulates: BET SSA measurements (https://doi.org/10.25375/uct.22309093.v1); characterization of dust-sized coal particulates: Malvern particle size analysis (https://doi.org/10.25375/uct.22308925.v1); characterization of dust-sized coal particulates: major element analysis using XRF; particle characterization, PLSR modeling parameters, and extensions of methodological descriptions (PDF)

## Abbreviations

CWP: coal workers’ pneumoconiosis
PLSR: partial least-squares regression
VIP: variable of importance
auto-SEM-EDS: automated scanning electron microscope coupled with energy-dispersive X-ray spectroscopy
QEMSCAN: quantitative evaluation of materials by scanning electron microscopy
XRD: X-ray diffraction
XRF: X-ray fluorescence
ECD: equivalent circular diameter
SSA: specific surface area
CRY: crystallite size
BET: Brunauer–Emmett–Teller
ROS: reactive oxygen species
MDA: malondialdehyde
LDH: lactate dehydrogenase

## References

[ref1] Ghose M. K.; Majee S. R. Assessment of the Impact on the Air Environment Due to Opencast Coal Mining - An Indian Case Study. Atmos. Environ. 2000, 34 (17), 2791–2796. 10.1016/S1352-2310(99)00302-7.

[ref2] Ghose M. K.; Majee S. R. Characteristics of Hazardous Airborne Dust around an Indian Surface Coal Mining Area. Environ. Monit. Assess. 2007, 130 (1–3), 17–25. 10.1007/s10661-006-9448-6. 17285255

[ref3] Huertas J. I.; Huertas M. E.; Cervantes G.; Díaz J. Assessment of the Natural Sources of Particulate Matter on the Opencast Mines Air Quality. Sci. Total Environ. 2014, 493 (2000), 1047–1055. 10.1016/j.scitotenv.2014.05.111. 25016110

[ref4] Yadav A. K.; Jamal A. Impact of Mining on Human Health in and around Mines. Environ. Qual. Manag. 2018, 28 (1), 83–87. 10.1002/tqem.21568.

[ref5] Shi P.; Xing X.; Xi S.; Jing H.; Yuan J.; Fu Z.; Zhao H. Trends in Global, Regional and National Incidence of Pneumoconiosis Caused by Different Aetiologies: An Analysis from the Global Burden of Disease Study 2017. Occup. Environ. Med. 2020, 77 (6), 407–414. 10.1136/oemed-2019-106321. 32188634

[ref6] Leonard R.; Zulfikar R.; Stansbury R. Coal Mining and Lung Disease in the 21st Century. Curr. Opin. Pulm. Med. 2020, 26 (2), 135–141. 10.1097/MCP.0000000000000653. 31815751

[ref7] Dalal N.; Newman J.; Pack D.; Leonard S.; Vallyathan V. Hydroxyl Radical Generation by Coal Mine Dust: Possible Implication to Coal Workers’ Pneumoconiosis (CWP). Free Radic. Biol. Med. 1995, 18 (1), 11–20. 10.1016/0891-5849(94)E0094-Y. 7896164

[ref8] Vallyathan V.; Schwegler D.; Reasor M.; Stettler L.; Clere J.; Green F. H. Y. Comparative *in Vitro* Cytotoxicity and Relative Pathogenicity of Mineral Dusts. Ann. Occup. Hyg. 1988, 32 (inhaled_particles_VI), 279–289. 10.1093/annhyg/32.inhaled_particles_VI.279.

[ref9] Zhang Q.; Dai J.; Ali A.; Chen L.; Huang X. Roles of Bioavailable Iron and Calcium in Coal Dust-Induced Oxidative Stress: Possible Implications in Coal Workers Lung Disease. Free Radic. Res. 2002, 36 (3), 285–294. 10.1080/10715760290019309. 12071347

[ref10] Cohn C. A.; Laffers R.; Simon S. R.; O’Riordan T.; Schoonen M. A. Role of Pyrite in Formation of Hydroxyl Radicals in Coal: Possible Implications for Human Health. Part. Fibre Toxicol. 2006, 3, 16 10.1186/1743-8977-3-16. 17177987 PMC1764420

[ref11] Huang X.; Gordon T.; Rom W. N.; Finkelman R. B. Interaction of Iron and Calcium Minerals in Coals and Their Roles in Coal Dust-Induced Health and Environmental Problems. Rev. Mineral. Geochem. 2006, 64 (1), 153–178. 10.2138/rmg.2006.64.6.

[ref12] Vallyathan V.; Shi X.; Castranova V. Reactive Oxygen Species: Their Relation to Pneumoconiosis and Carcinogenesis. Environ. Health Perspect. 1998, 106, 1151–1155. 10.2307/3433978. 9788890 PMC1533374

[ref13] Schins R. P. F.; Borm P. J. A. Mechanisms and Mediators in Coal Dust Induced Toxicity: A Review. Ann. Occup. Hyg. 1999, 43, 7–33. 10.1016/S0003-4878(98)00069-6. 10028891

[ref14] Sun Y.; Kinsela A. S.; Waite T. D. Elucidation of Alveolar Macrophage Cell Response to Coal Dusts: Role of Ferroptosis in Pathogenesis of Coal Workers’ Pneumoconiosis. Sci. Total Environ. 2022, 823, 153727 10.1016/j.scitotenv.2022.153727. 35149061

[ref15] Lison D.; Lardot C.; Huaux F.; Zanetti G.; Fubini B. Influence of Particle Surface Area on the Toxicity of Insoluble Manganese Dioxide Dusts. Arch. Toxicol. 1997, 71, 725–729. 10.1007/s002040050453. 9388004

[ref16] Champion J. A.; Mitragotri S. Role of Target Geometry in Phagocytosis. Proc. Natl. Acad. Sci. U.S.A. 2006, 103 (13), 4930–4934. 10.1073/pnas.0600997103. 16549762 PMC1458772

[ref17] Fubini B.; Fenoglio I. Toxic Potential of Mineral Dusts. Elements 2007, 3 (6), 407–414. 10.2113/GSELEMENTS.3.6.407.

[ref18] Turci F.; Pavan C.; Leinardi R.; Tomatis M.; Pastero L.; Garry D.; Anguissola S.; Lison D.; Fubini B. Revisiting the Paradigm of Silica Pathogenicity with Synthetic Quartz Crystals: The Role of Crystallinity and Surface Disorder. Part. Fibre Toxicol. 2015, 13, 32 10.1186/s12989-016-0136-6. PMC4902968 27286702

[ref19] Sun Y.; Kinsela A. S.; Cen X.; Sun S.; Collins R. N.; Cliff D. I.; Wu Y.; Waite T. D. Impact of Reactive Iron in Coal Mine Dust on Oxidant Generation and Epithelial Lung Cell Viability. Sci. Total Environ. 2022, 810, 152277 10.1016/j.scitotenv.2021.152277. 34902414

[ref20] Gormley I. P.; Collings P.; Davis J. M. G.; Ottery J. An Investigation into the Cytotoxicity of Respirable Dusts from British Collieries. Br. J. Exp. Pathol. 1979, 60, 526–536. 518822 PMC2041501

[ref21] Reisner M. T. R.; Bruch J.; Hilscher W.; Kriegseis W.; Prajsnar D.; Robock K.; Rosmanith J.; Scharmann A.; Schlipköter H. W.; Strübel G.; Weller W. Specific Harmfulness of Respirable Dusts from West German Coal Mines VI: Comparison of Experimental and Epidemiological Results. Ann. Occup. Hyg. 1982, 26 (4), 527–539. 10.1093/annhyg/26.4.527. 7181288

[ref22] Trechera P.; Moreno T.; Córdoba P.; Moreno N.; Zhuang X.; Li B.; Li J.; Shangguan Y.; Kandler K.; Dominguez A. O.; Kelly F.; Querol X. Mineralogy, Geochemistry and Toxicity of Size-Segregated Respirable Deposited Dust in Underground Coal Mines. J. Hazard. Mater. 2020, 399, 122935 10.1016/j.jhazmat.2020.122935. 32540702

[ref23] Song Y.; Southam K.; Basil B.; Zosky G. R.; Graeme Zosky C. R.; Bardin P.; Reynolds P. Effects of Chemical Composition on the Lung Cell Response to Coal Particles: Implications for Coal Workers’ Pneumoconiosis. Respirology 2022, 12, 447–454. 10.1111/resp.14246. PMC9314662 35306722

[ref24] Kamanzi C.; Becker M.; Jacobs M.; Konečný P.; Von Holdt J.; Broadhurst J. The Impact of Coal Mine Dust Characteristics on Pathways to Respiratory Harm: Investigating the Pneumoconiotic Potency of Coals. Environ. Geochem. Health 2023, 45, 7363–7388. 10.1007/s10653-023-01583-y. 37131112 PMC10517901

[ref25] Trechera P.; Moreno T.; Córdoba P.; Moreno N.; Zhuang X.; Li B.; Li J.; Shangguan Y.; Dominguez A. O.; Kelly F.; Querol X. Comprehensive Evaluation of Potential Coal Mine Dust Emissions in an Open-Pit Coal Mine in Northwest China. Int. J. Coal Geol. 2021, 235, 103677 10.1016/j.coal.2021.103677.

[ref26] Trechera P.; Moreno T.; Córdoba P.; Moreno N.; Amato F.; Cortés J.; Zhuang X.; Li B.; Li J.; Shangguan Y.; Dominguez A. O.; Kelly F.; Mhadhbi T.; Jaffrezo J. L.; Uzu G.; Querol X. Geochemistry and Oxidative Potential of the Respirable Fraction of Powdered Mined Chinese Coals. Sci. Total Environ. 2021, 800, 149486 10.1016/j.scitotenv.2021.149486. 34391157

[ref27] Shangguan Y.; Zhuang X.; Querol X.; Li B.; Moreno N.; Trechera P.; Sola P. C.; Uzu G.; Li J. Characterization of Deposited Dust and Its Respirable Fractions in Underground Coal Mines: Implications for Oxidative Potential-Driving Species and Source Apportionment. Int. J. Coal Geol. 2022, 258, 104017 10.1016/j.coal.2022.104017.

[ref28] Kamanzi C.; Becker M.; Von Holdt J.; Broadhurst J. Development of a SEM-EDS-XRD Protocol for the Physicochemical and Automated Mineralogical Characterisation of Coal Dust Particles. Resources 2022, 11, 114 10.3390/resources11120114.

[ref29] Lee C. Y.; Lee S. L.; Sheehan C. E.; Wang Y. Composition of Coal Dusts and Their Cytotoxicity on Alveolar Macrophages; Technical Report ARCCB-TR-96026; Cambridge University Press, 1996, accessed 2019–05–10. https://pdfs.semanticscholar.org/8185/85459224e1ac9db8dce8b006c415033ec7d9.pdf.

[ref30] van Maanen J. M. S.; Borm P. J. A.; Knaapen A.; van Herwijnen M.; Schilderman P. A.; Smith K. R.; Aust A. E.; Tomatis M. *In Vitro* Effects of Coal Fly Ashes: Hydroxyl Radical Generation, Iron Release, and DNA Damage and Toxicity in Rat Lung Epithelial Cells. Inhal. Toxicol. 1999, 11, 1123–1141. 10.1080/089583799196628. 10562700

[ref31] Beamish B.; Zosky G. Assessment of Pyritie Coal Dust Induced Pneumoconiosis; ACARP, 2019.

[ref32] Abdi H. Partial Least Squares Regression and Projection on Latent Structure Regression (PLS Regression). WIREs Comp. Stats. 2010, 2 (1), 97–106. 10.1002/wics.51.

[ref33] Mevik B.-H.; Wehrens R. The Pls Package: Principal Component and Partial Least Squares Regression in R. J. Stat. Software 2007, 18 (2), 1–24. 10.18637/jss.v018.i02.

[ref34] Mevik B.-H. Introduction to the Pls Package; CRAN: Netherlands, 2022; pp 1–24, accessed 2022–12–12. https://cran.r-project.org/web/packages/pls/vignettes/pls-manual.pdf.

[ref35] De Jong S. SIMPLS: An alternative approach to partial least squares regression. Chemometr. Intell. Lab. Syst. 1993, 18, 251–263. 10.1016/0169-7439(93)85002-X.

[ref36] Vallyathan V. Generation of Oxygen Radicals by Minerals and Its Correlation to Cytotoxicity. Environ. Health Perspect. 1994, 102, 111–115. 10.1289/ehp.94102s10111. PMC1566993 7705284

[ref37] Zhang Q.; Huang X. Induction of Ferritin and Lipid Peroxidation by Coal Samples With Different Prevalence of Coal Workers’ Pneumoconiosis: Role of Iron in the Coals. Am. J. Ind. Med. 2002, 42, 171–179. 10.1002/ajim.10101. 12210686

[ref38] Harrington A. D.; Tsirka S. E.; Schoonen M. A. A. Inflammatory Stress Response in A549 Cells as a Result of Exposure to Coal: Evidence for the Role of Pyrite in Coal Workers’ Pneumoconiosis Pathogenesis. Chemosphere 2013, 93 (6), 1216–1221. 10.1016/j.chemosphere.2013.06.082. 23895739 PMC3957027

[ref39] Orona N. S.; Astort F.; Maglione G. A.; Saldiva P. H. N.; Yakisich J. S.; Tasat D. R. Direct and Indirect Air Particle Cytotoxicity in Human Alveolar Epithelial Cells. Toxicol. Vitro 2014, 28, 796–802. 10.1016/j.tiv.2014.02.011. 24590061

[ref40] Yang H.; Liu C.; Yang D.; Zhang H.; Xi Z. Comparative study of cytotoxicity, oxidative stress and genotoxicity induced by four typical nanomaterials: the role of particle size, shape and composition. J. Appl. Toxicol. 2009, 29, 69–78. 10.1002/jat.1385. 18756589

[ref41] Champion J. A.; Katare Y. K.; Mitragotri S. Particle Shape: A New Design Parameter for Micro- and Nanoscale Drug Delivery Carriers. J. Controlled Release 2007, 121 (1–2), 3–9. 10.1016/j.jconrel.2007.03.022. PMC4009069 17544538

[ref42] O’Neill L. A. J. How Frustration Leads to Inflammation. Science 2008, 320, 619–620. 10.1126/science.1158398. 18451288

[ref43] Kass G.; Orrenius S. Calcium Signaling and Cytotoxicity. Environ. Health Perspect. 1999, 107, 25–35. 10.2307/3434469. 10229704 PMC1566353

[ref44] Donaldson K.; Stone V.; Borm P. J. A.; Jimenez L. A.; Gilmour P. S.; Schins R. P. F.; Knaapen A. M.; Rahman I.; Faux S. P.; Brown D. M.; MacNee W. Oxidative Stress and Calcium Signaling in the Adverse Effects of Environmental Particles (PM10). Free Radic. Biol. Med. 2003, 34 (11), 1369–1382. 10.1016/S0891-5849(03)00150-3. 12757847

[ref45] Dolmetsch R.; Xu K.; Lewis R. S. Calcium Oscillations Increase the Efficiency and Specificity of Gene Expression. Nature 1998, 392, 933–936. 10.1038/31960. 9582075

[ref46] Dolmetsch R. E.; Lewis R. S.; Goodnow C. C.; Healy J. I. Differential Activation of Transcription Factors Induced by Ca2+ Response Amplitude and Duration. Nature 1997, 386 (6627), 855–858. 10.1038/386855a0. 9126747

[ref47] Warr L. N.; Nieto F. Crystallite Thickness and Defect Density of Phyllosilicates in Low-Temperature Metamorphic Pelites: A TEM and XRD Study of Clay-Mineral Crystallinity-Index Standards. Can. Mineral. 1998, 36 (6), 1453–1474.

[ref48] Kongsuebchart W.; Praserthdam P.; Panpranot J.; Sirisuk A.; Supphasrirongjaroen P.; Satayaprasert C. Effect of Crystallite Size on the Surface Defect of Nano-TiO2 Prepared via Solvothermal Synthesis. J. Cryst. Growth 2006, 297 (1), 234–238. 10.1016/j.jcrysgro.2006.09.018.

[ref49] Davies R. Factors Involved in the Cytotoxicity of Kaolinite towards Macrophages *in Vitro*. Environ. Health Perspect. 1983, 51, 249–252. 10.1289/ehp.8351249. 6641658 PMC1569275

[ref50] Davies R.; Griffiths D. M.; Johnson N. F.; Preece A. W.; Livingston D. C. The Cytotoxicity of Kaolin towards Macrophages *in Vitro*. Br. J. Exp. Pathol. 1984, 65, 453–466. 6466554 PMC2040988

[ref51] Wastiaux A.; Daniel H. Pulmonary Toxicity of Kaolin in Rats Exposed by Inhalation. Health Related Effects of Phyllosilicates; Springer, 1990, pp 405–414.

[ref52] Weaver C. E. The Nature of TiO2 in Kaolinite. Clays Clay Miner. 1976, 24 (5), 215–218. 10.1346/CCMN.1976.0240501.

[ref53] Mu M.; Li B.; Zou Y.; Wang W.; Cao H.; Zhang Y.; Sun Q.; Chen H.; Ge D.; Tao H.; Hu D.; Yuan L.; Tao X.; Wang J. Coal Dust Exposure Triggers Heterogeneity of Transcriptional Profiles in Mouse Pneumoconiosis and Vitamin D Remedies. Part. Fibre Toxicol. 2022, 19 (1), 7–21. 10.1186/s12989-022-00449-y. 35057792 PMC8772169

[ref54] Zhang Y.; Liang J.; Cao N.; Gao J.; Song L.; Tang X. Coal dust nanoparticles induced pulmonary fibrosis by promoting inflammation and epithelial-mesenchymal transition via the NF-κB/NLRP3 pathway driven by IGF1/ROS-mediated AKT/GSK3β signals. Cell Death Discovery 2022, 8 (1), 500 10.1038/s41420-022-01291-z. 36581638 PMC9800584

[ref55] Zhang Y.; Li A.; Gao J.; Liang J.; Cao N.; Zhou S.; Tang X. Differences in the Characteristics and Pulmonary Toxicity of Nano- and Micron-Sized Respirable Coal Dust. Respir. Res. 2022, 23 (1), 197 10.1186/s12931-022-02120-8. 35906696 PMC9338665

