# Supplementary material for: Machine Learning Demonstrates Dominance of Physical Characteristics over Particle Composition in Coal Dust Toxicity
Source: Environ Sci Technol. 2024 Jan 8;58(3):1636–47. doi: 10.1021/acs.est.3c08732 (PMC10809749; doi:10.1021/acs.est.3c08732)
Supplement: Supplementary file 1 — es3c08732_si_001.pdf [file es3c08732_si_001.pdf]

Machine learning demonstrates dominance of physical characteristics over particle composition in coal dust toxicity

Conchita Kamanzi[*,†,1,2]; *Megan Becker*[1,2]; *Johanna Von Holdt*[3], *Nai-Jen Hsu*[4]; *Petr Konečný*[4], *Jennifer Broadhurst*[1]; *Muazzam Jacobs*[4,5,6]

[1]Department of Chemical Engineering, Minerals to Metals initiative, University of Cape Town, Cape Town, South Africa

[2]Department of Chemical Engineering, Centre for Minerals Research, University of Cape Town, Cape Town, South Africa

[3]Department of Environmental and Geographical Science, University of Cape town, Cape Town, South Africa

[4]Welcome Centre for Infectious Diseases Research in Africa, Institute for Infectious Diseases and Molecular Medicine, Division of Immunology, Department of Pathology, University of Cape Town, Cape Town, South Africa

[5]Neuroscience Institute, University of Cape Town, Cape Town, South Africa

[6]National Health Laboratory Service, Johannesburg, South Africa

Corresponding author information:
[*]Conchita Kamanzi
Email: Conchita.Kamanzi@uct.ac.za

This PDF file includes pages S1-S20:

Figures S1 to S7
Tables S1 to S8
Sections S1 to 4
SI References

Other supporting materials for this manuscript include the following:

Datasets S1 to S5

Supplementary Figures

Fig. S1. Variation between the mineral abundances defined by X-Ray Diffraction (XRD) and QEMSCAN. Figures A, B, and C represent the comparison of the major phases ranging between 0-100 mass % abundance respectively. Figures D, E, and F represent the comparison of the minor phases ranging between 0-40 mass % abundance respectively.

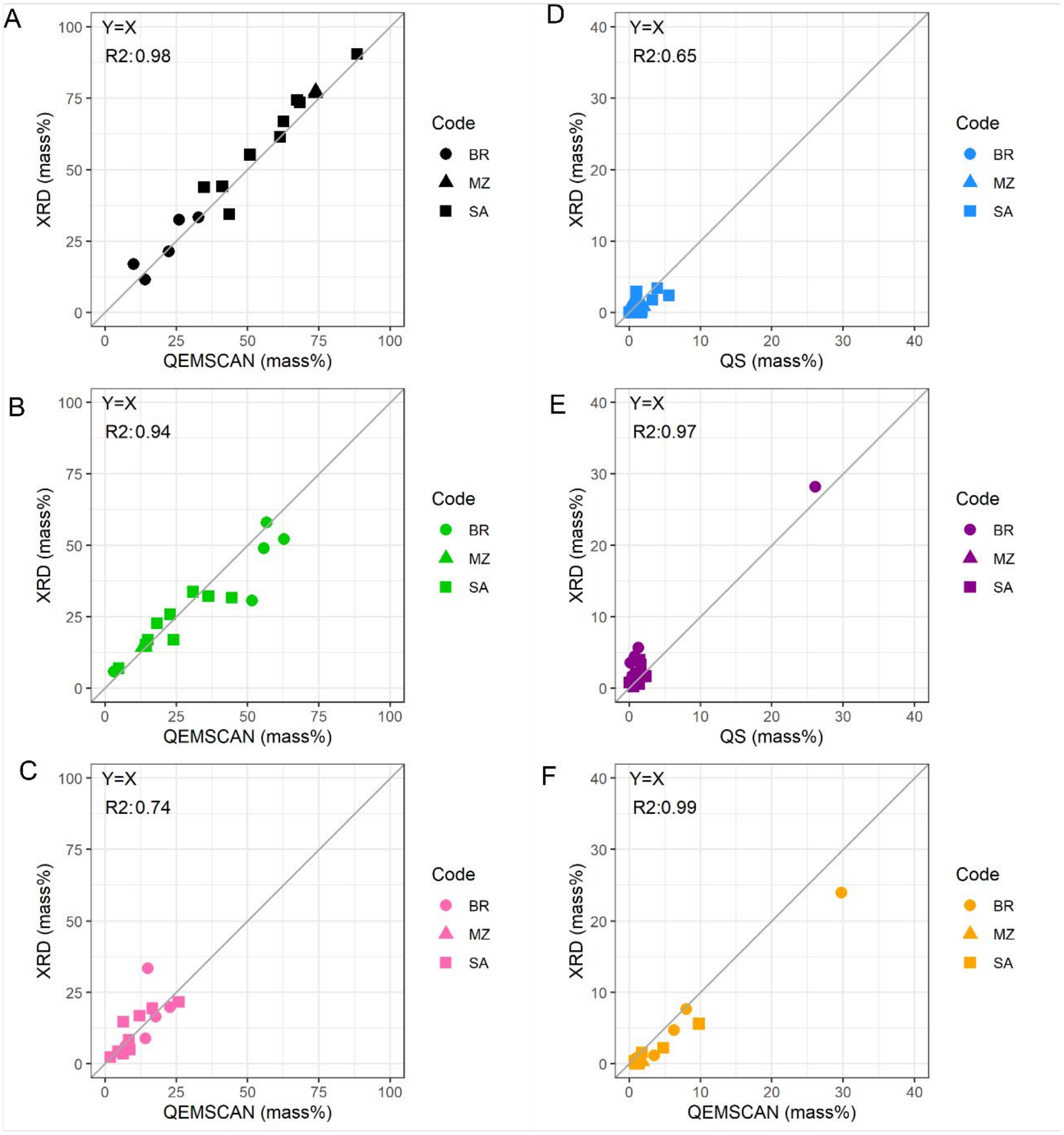

Fig. S2. Distribution of the particle roughness and shape classes amongst the samples investigated. A and B represent the range in the percentage abundance of the roughness and shape classes amongst the samples analysed respectively using boxplots. The false colour particle images represent examples of the shapes and roughness classed by the QEMSCAN auto-SEM.

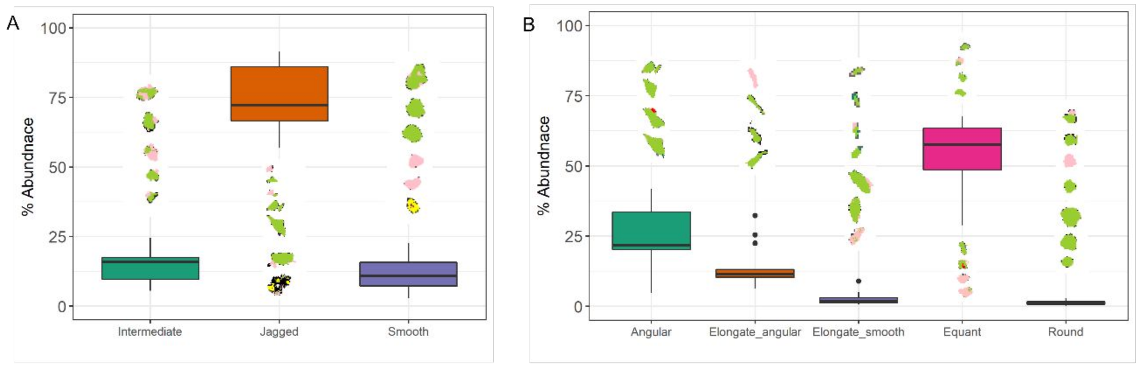

Fig. S3. Graphical false colour description of the liberation classes considering quartz (pink) as the mineral of interest (MOI). The bar graphs on the right hand side of the figure show an example of the relative association of quartz with additional phases (clays = green and carbonaceous matter = black). In terms of their definitions the liberation categories were classed as: liberated = % Area MOI ≤ 100 and% Area MOI ≥ 70, moderately liberated = % Area MOI < 70 and% Area MOI ≥ 40, mostly encapsulated = % Area MOI < 40 and% Area MOI ≥ 10, fully encapsulated = % Area MOI < 10 and% Area MOI ≥ 0.

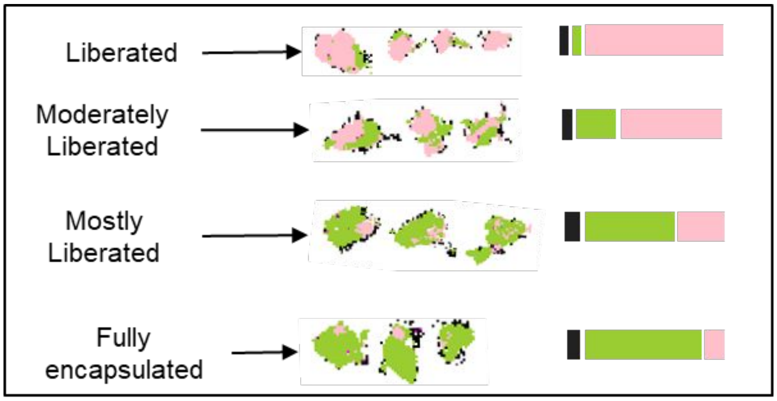

Fig. S4. The relationship between mineral matter and SSA across the locations of origin.

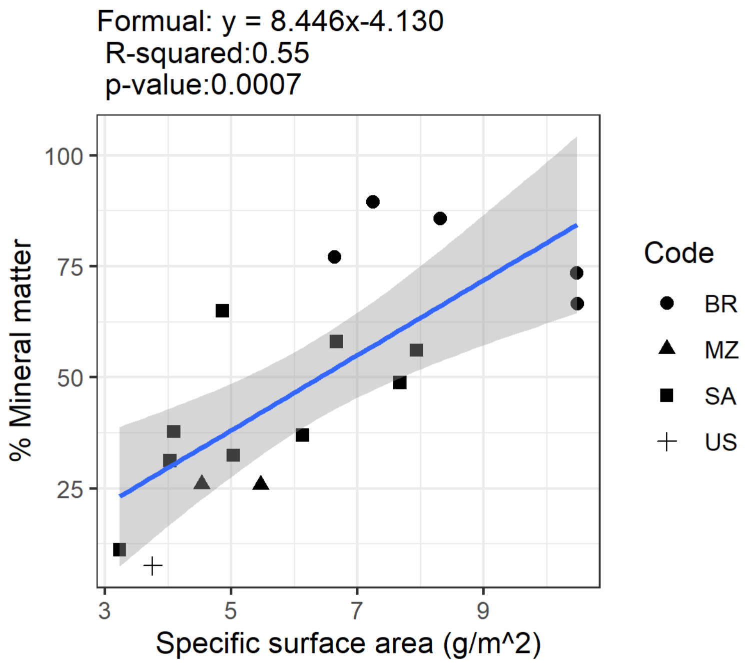

Fig. S5. Distribution of the crystallite size reported amongst the minerals kaolinite, pyrite, and quartz. Individual panels depict the distribution and range of crystallite sizes for either kaolinite, pyrite, or quartz, grouped by the country of origin (BR-Brazil, MZ-Mozambique, SA-South Africa, US-USA). The values were calculated from XRD diffractograms using the Scherrer equation in the Rietveld method[1].

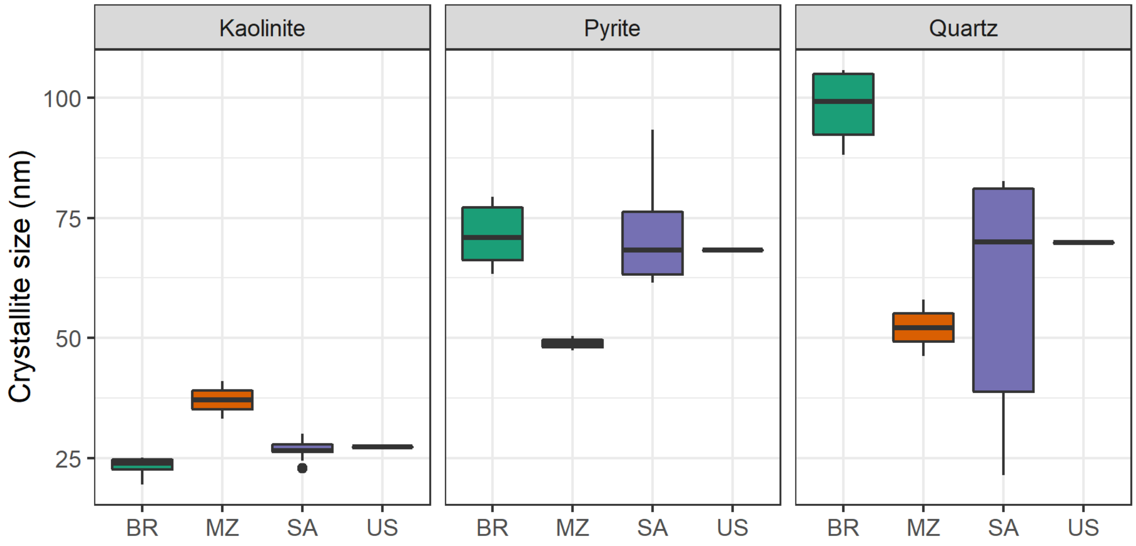

Fig. S6. Degree of liberation amongst particles containing quartz, pyrite, or clays across the 17 samples investigated.

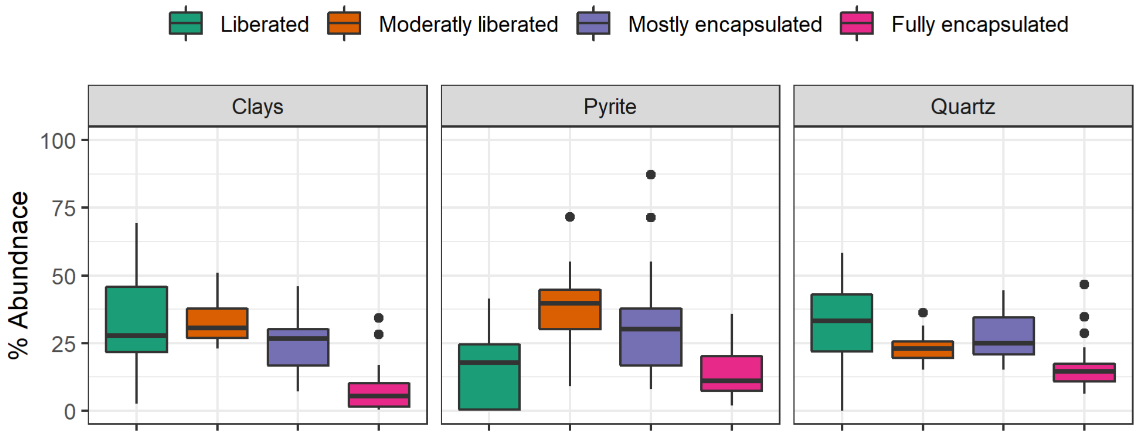

Fig. S7. Description of titanium accountability by minerals hosts. A and B represent the relationship between the abundance of clays and rutile (defined by the Quantitative Evaluation of Materials by Scanning Electron Microscopy (QEMSCAN) auto-Scanning Electron Microscope) to the total assayable titanium content respectively.

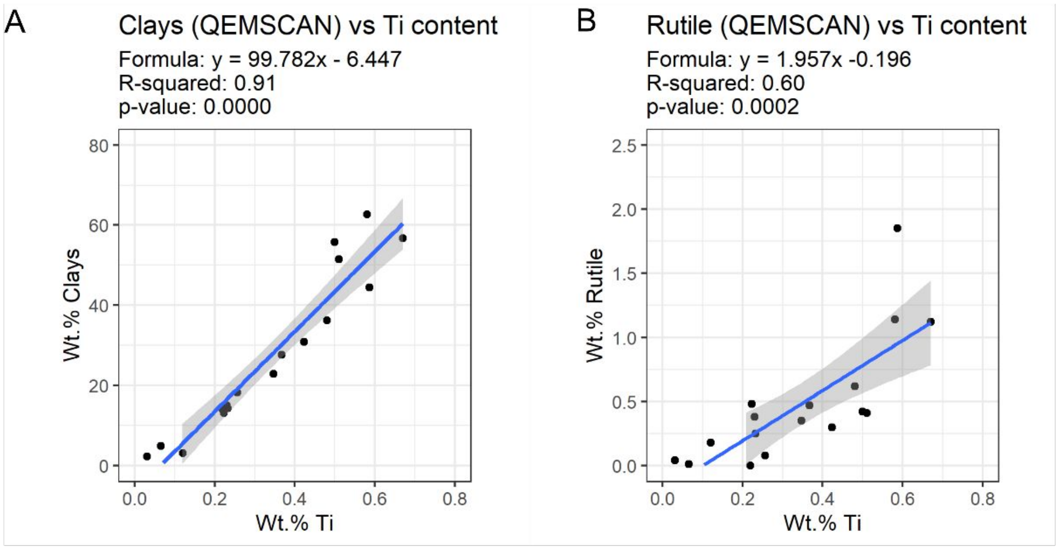

Supplementary Tables

Table S1. Description of samples presented in the study and the D50 representing the median particle size determined with the Malvern Mastersizer 2000 (Pananalytical).

Sample name	Description	D50 (μm)
SA-Dis1*	South African discard (mine G Waterberg coal field)	-
SA -UF1	South African ultrafine thickener underflow (mine G Waterberg coal field)	11.02
SA -MDT	South African medium dense (mine L Witbank coal field)	8.49
SA -Dis2	South African discard (mine G Witbank coal field)	9.73
SA -UF2	South African ultrafine thickener underflow (mine L Witbank coal field)	9.53
SA -UF3*	South African ultrafine (mine G Witbank coal field)	-
SA -UF4	South African ultrafine (mine P Witbank coal field)	9.78
SA -ROM3	South African run of mine (Sasolburg coal field)	9.66
SA -ROM2*	South African run of mine (Witbank coal field)	-
Br-MDT	Brazilian coal medium dense (Santa Catarina coal field)	7.18
Br-DSulf	Brazilian coal desulfurised (Santa Catarina coal field)	8.85
Br-PyC	Brazilian coal high pyrite concentrate (Santa Catarina coal field)	10.42
Br-Tail	Brazilian coal dump (Santa Catarina coal field)	8.36
Br-Dis	Brazilian coal discards (Santa Catarina coal field)	7.70
Mz-ROM1*	Mozambican coal run of mine (Moatize coal field)	-
Mz-ROM2*	Mozambican coal run of mine (Moatize coal field)	-
US-ROM*	USA coal run of mine (Pittsburgh coal field)	-

* For coals that displayed hydrophobic properties, no reading could be collected

Table S2. Particle size distribution and mean size of the particles reported as Equivalent Circular Diameter (ECD). The distribution data represents the percentage mass of the total population in the size range of 5 to 25 μm in increments of 5 μm. The results were obtained through the analysis of particle cross sections using the QEMSCAN auto-SEM system.

Sample	N*	< 5 μm	< 10 μm	< 15 μm	< 20 μm	< 25 μm	Mean ECD (μm)
Br-Dsulf	3799	12.6	30.5	25.7	21.3	9.8	11.8
Br-Dis	3334	2.9	16.9	31.9	31.4	16.9	14.6
Br-Tail	3009	10.6	30.8	28.2	22.6	8.0	11.8
Br-MDT	4330	30.2	34.7	18.0	12.5	4.6	8.8
SA-UF1	2811	11.3	24.2	27.5	25.9	11.1	12.6
SA-Dis2	40042	19.7	28.2	23.6	17.8	10.7	11.1
SA-MDT	1193	14.3	37.3	25.5	17.1	6.0	10.7
SA-UF2	1262	22.9	36.9	24.3	11.5	4.4	9.4
Br-PyC	4675	8.8	24.9	25.6	23.2	17.6	13.3
Mz-ROM1	1144	23.5	36.9	16.7	14.5	8.4	9.9
Mz-ROM2	1175	25.5	32.4	25.6	11.3	5.1	9.4
US-ROM	13238	15.4	36.4	27.9	17.6	2.6	10.3
SA-ROM2	2453	24.1	35.0	24.6	13.4	2.9	9.3
SA-ROM3	3435	11.0	18.1	23.6	29.7	17.6	13.7
SA-UF3	2660	16.0	34.4	22.9	17.7	9.0	11.0
SA-UF4	15929	24.9	39.5	21.8	11.7	2.2	8.8
SA-Dis1	3858	29.1	32.5	20.8	11.0	6.7	9.2

*N – number of particles analysed

Table S3. Mineral distribution for each sample determined by quantitative XRD analysis.

Sample	CM	Qz	Kln	Ilt	Pl	Py	Cal	Dol	Gp	Szo	Rt	Rbc
Br-Dsulf	11.5	19.9	35.9	22.4	2.6	1.2	-	-	4.4	-	-	-
Br-Dis	16.9	16.5	27.8	24.3	2.4	4.7	1.5	-	1.7	-	1.8	-
Br-Tail	21.4	33.4	25.7	4.9	-	7.6	-	-	5.7	-	1.2	-
Br-MDT	32.5	8.9	28.4	20.5	2.7	0.8	1.1	-	2.2	-	-	1.3
SA-UF1	44.1	21.7	18.8	3.9	-	0.4	2.4	5.5	1.6	-	0.6	-
SA-Dis2	34.4	16.8	25.2	8.5	-	5.6	2.9	-	3.3	-	2.2	-
SA-MDT	43.8	19.4	29.9	2.7	-	1.6	-	-	-	-	2.0	-
SA-UF2	55.2	8.3	27.4	4.8	-	0.5	-	-	1.4	-	2.3	-
Br-PyC	33.3	6.3	5.8	-	-	24.0	0.2	-	0.7	6.6	-	19.3
Mz-ROM1	76.0	5.2	8.8	5.5	-	0.2	0.8	0.5	0.1	1.4	-	-
Mz-ROM2	75.2	5.2	8.7	5.6	-	0.3	1.4	0.4	1.0	1.8	0.1	-
*US-ROM	-	-	-	-	-	-	-	-	-	-	-	-
SA-ROM2	89.7	2.3	6.9	0.8	-	-	-	0.4	0.3	0.2	-	-
SA-ROM3	64.5	5.0	25.8	-	-	-	0.1	0.3	0.1	1.9	-	-
SA-UF3	73.6	4.3	12.3	2.8	-	0.4	3.4	1.5	0.5	-	1.4	-
SA-UF4	74.4	3.5	13.7	3.2	-	0.4	1.7	1.1	0.7	-	1.3	-
SA-Dis1	61.5	14.8	17.0	-	-	2.2	-	-	2.3	-	0.7	0.7

*For sample US-ROM the amorphous content was too high to perform the XRD refinement.

CM-carbonaceous matter estimated from the ash content normalised to the total contribution of volatile compounds in each coal

Table S4. Description of samples presented in the study and the D50 representing the median particle size determined with the Malvern Mastersizer 2000 (Pananalytical).

		Cross-validation results		% Explained variability		Model estimation
	n Comps	RMSEP (CV)	RMSEP (adjCV)	Explanatory variable (X)	Response variable (Y)	R^2 (Predictions vs Observations)
LDH	1	0.8489	0.8240	19.94	71.49	0.86
	2	0.8305	0.7997	32.33	85.70	0.95
	**3**	**0.8147**	**0.7811**	**46.69**	**91.18**	**0.95**
MDA	1	0.9921	0.9361	21.86	79.56	0.88
	2	0.898	0.842	36.95	93.28	0.96
	3	0.8257	0.7718	52.93	97.08	0.97
	**4**	**0.7736**	**0.7236**	**63.71**	**98.26**	**0.99**

Table S5. Mineral group and elemental composition for the full list of minerals identified by the QEMSCAN auto-SEM.

Mineral Name	Mineral group	Composition (% element)
Kaolinite	Silicate	Al 20.90, H 1.56, O 55.78, Si 21.76
Quartz	Silicate	Si 46.74, O 53.26
Muscovite	Silicate	Al 20.30, F 0.95, H 0.46, K 9.81, O 47.35, Si 21.13
Zircon	Silicate	Zr 43.14, Hf 4.69, La 3.78, Si 14.76, O 33.63
Illite	Silicate	Al 9.01, Fe 1.43, H 1.35, K 6.03, Mg 1.87, O 55.06, Si 25.25
Talc	Silicate	Mg 19.23, H 0.53, O 50.62, Si 29.62
Szomolnokite	Sulfate	Fe 32.87, H 1.19, O 47.07, S 18.87
Rhomboclase	Sulfate	Fe 17.40, H 2.83, O 59.80, S 19.97
Alunogen	Sulfate	Al 8.32, H 5.29, O 71.55, S 14.84
Coquimbite	Sulfate	Fe 19.87, H 3.23, O 59.78, S 17.12
Voltaite	Sulfate	Al 1.33, Fe 22.02, H 1.79, K 3.85, O 52.04, S 18.96
Hydronium-jarosite	Sulfate	Fe 34.85, H 1.89, O 49.92, S 13.34
Jarosite	Sulfate	Fe 33.45, H1.21, K 7.81, O 44.72, S 12.81
Gypsum	Sulfate	Ca 23.28, H 2.34, O 55.76, S 18.62
Dolomite	Carbonate	Ca 21.73, Mg 13.18, C 13.03, O 52.06
Siderite	Carbonate	Fe 48.20, C 10.37, O 41.43
Ankerite	Carbonate	Ca 19.42, Fe 16.24, Mg 3.53, Mn 2.66, C 11.64, O 46.51
Calcite	Carbonate	Ca 40.04, C 12.00, O 47.96
Apatite	Phosphate	Ca 39.36, Cl 2.32, F 1.24, H 0.07, O 38.76, P 18.25
Pyrite	Sulfide	Fe 46.55, S 53.45
Pyrrhotite	Sulfide	Fe 62.33, S 37.67
Barite	Sulfide	Ba 58.84, O 27.42, S 13.74
Galena	Sulfide	Pb 86.60, S 13.40
Sphalerite	Sulfide	Fe 2.88, S 33.06, Zn 64.06

Chalcopyrite	Sulfide	Cu 34.63, Fe 30.43, S 34.94
Molybdenite	Sulfide	Mo 59.94, S 40.06
Bornite	Sulfide	Cu 63.31, Fe 11.13, S 25.56
Rutile	Oxide	Ti 59.94, O 40.06
Spinel	Oxide	Al 37.93, Mg 17.08, O 44.98
Hematite	Oxide	Fe 69.94, O 30.06
Goethite	Hydroxide	Fe 62.85, H 1.13, O 36.01
Gibbsite	Hydroxide	Al 34.59, H 3.88, O 61.53

Table S6. Multilinear analysis of the relationship between the proportion of fines generated from a sample and the total abundance of carbonaceous matter and clays in each sample.

Parameter	Value	Estimate p-value relative to model
Coefficient (Carbonaceous matter)	0.270	0.0339
Coefficient (Clays)	0.203	0.1821
R-squared	0.32	
Overall model p-value	0.0687	

Table S7. Outputs from the linear regression applied to model the dose-response relationships per sample. The goodness of fit was determined by the adjusted R2, coefficient representing the gradient of the straight line, standard error defining the uncertainty in the model, and the strength of the relationship defined by the p-value (p <0.05 statistically significant, p <0.01 highly significant).

Sample	Adjusted R^2	Coefficient	Standard error	p-value
Br-Dsulf	0.99	0.085	0.006	0.004
Br-Dis	0.87	0.057	0.012	0.044
*Br-Tail	0.80	0.037	0.010	0.067
Br-PyC	1.00	0.088	0.004	0.002
Br-MDT	0.99	0.071	0.003	0.002
Mz-ROM1	0.99	0.031	0.001	0.002
Mz-ROM2	0.98	0.038	0.003	0.008
US-ROM	0.92	0.025	0.004	0.027
SA-ROM2	0.84	0.025	0.006	0.056
SA-ROM3	0.98	0.049	0.004	0.005
SA-Dis1	0.98	0.097	0.009	0.008
SA-UF1	0.91	0.052	0.010	0.032
SA-Dis2	0.99	0.073	0.005	0.005
SA-UF3	0.92	0.040	0.007	0.026
SA-MDT	0.92	0.056	0.009	0.027
SA-UF2	0.98	0.067	0.005	0.005
SA-UF4	0.91	0.040	0.007	0.032

*Coefficient was still considered irrespective of the p-value (0.067) as the gradient was still considered reasonable compared to a sample with an overlapping fit (Br-Dis)

Table S8. Mass percentage of particles reporting to the different liberation classes, further grouped by level of MDA release.

MDA level	Samples	Liberated quartz (mass %)	Moderately liberated quartz (mass %)	Mostly encapsulated quartz (mass %)	Fully encapsulated quartz (mass %)
H	Br-PyC	30.72	19.42	32.64	17.22
H	SA-Dis1	20.69	19.91	35.89	23.51
Level average		**25.70 ± 7.09**	**19.66 ± 0.34**	**34.27 ± 2.30**	**20.36 ± 4.45**
M	Br-Dsulf	58.41	15.16	19.54	6.92
M	Br-MDT	28.35	25.04	33.57	13.05
M	SA-UF1	27.88	31.39	34.52	6.22
M	SA-Dis2	37.88	23.41	24.98	13.65
M	SA-MDT	35.90	36.14	20.81	7.22
M	SA-UF2	45.92	17.75	21.34	14.99
Level average		**39.06 ± 11.60**	**24.81 ± 7.96**	**25.79 ± 6.65**	**10.34 ± 3.96**
L	Br-Dis	21.96	29.95	36.20	11.88
L	SA-ROM2	0.00	25.65	28.27	46.60
L	SA-ROM3	33.26	15.18	23.06	28.51
L	SA-UF3	40.57	22.59	20.83	16.23
L	SA-UF4	57.73	17.98	15.14	9.15
Level average		**30.70 ± 21.53**	**22.27 ± 5.90**	**24.70 ± 7.97**	**22.47 ± 15. 39**

Supplementary sections

Section S1: Additional detail on X-ray Diffraction methodology

The measurements were conducted using a Malvern Panalytical Aeris diffractometer with a PiXcel detector and fixed slits with Fe-filtered Co-Kα radiation. A randomly ordered powder mount of the sample was prepared for analysis using the back-loading method. Diffraction patterns were subsequently measured in the scan range 5.0000–80.0002° at 2Ɵ, with a step size 0.0217° at a count time of 48.195 s per step. To identify and quantify the crystalline phases from the diffractogram, the Bruker DIFFRAC.EVA and TOPAS software was used by applying the Rietveld refinement method[2].

Section S2: Additional detail on X-ray Fluorescence methodology

The samples were crushed to a fine powder (particle size < 70 μm) with a jaw crusher and milled in a tungsten-carbide ring mill prior to the preparation of a fused disc for major and trace elements analysis. The jaw crusher and mill were cleaned with clean uncontaminated quartz between samples to avoid cross contamination. Glass disks were prepared for XRF analysis using 7 g of high purity trace element and Rare Earth Element free flux (LiBO2 = 32.83%, Li2B4O7 = 66.67%, LiI = 0.50%) mixed with 0.7g of the powder sample. A mixture of sample and flux were fused in platinum crucibles with a Claisse M4 gas fluxer at temp between 1100°C–1200°C.

Whole-rock major element compositions were determined by XRF spectrometry on a PANalytical Axios Wavelength Dispersive spectrometer. The samples were crushed to a fine powder (particle size < 70 μm) with a jaw crusher and milled in a tungsten-carbide ring mill prior to the preparation of a fused disc for major and trace elements analysis. The jaw crusher and mill were cleaned with clean uncontaminated quartz between samples to avoid cross contamination. Whole-rock major element compositions were analysed on the fused glass disk using a 2.4 kW Rhodium tube. Matrix effects in the samples were corrected by applying theoretical alpha factors and measured line overlap factors to the raw intensities measured with the SuperQ PANalytical software.

Section S3: Additional detail on BET analysis crystallite size analysis

To measure specific surface area, a Micromeritics Tristar II 3020 instrument was used to measure the amount of adsorbed nitrogen corresponding to a monomolecular layer on the surface of the sample. To ensure that no absorbed gasses or vapours are present on the sample through handling, preparations were made by "outgassing" before the measurement at 120°C for 12 hours.

Section S4: Additional detail on crystallite size analysis

Crystallite size was analysed to measure the smallest single crystal in a powdered form and by extension represent the level of surface defects between minerals. X-ray Diffraction (XRD) is commonly used to obtain an estimate of crystallite size for each mineral represented in the diffractogram[1]. To determine estimates of the crystallite size of target minerals, the XRD diffractograms were processed in the Bruker software TOPAS using the Scherrer equation in the Rietveld method[1].

Section S5: further discussion on the effect of gypsum hosted Ca on cellular damage

Ca derived from calcite and dolomite were found to have a strong negative impact on lipid peroxidation while Ca derived from gypsum strongly and positively influenced the extent lipid peroxidation. In relation to the known interactions between calcite, pyrite and their combined product gypsum, the basis of explaining the negative impact of Ca derived from calcite and dolomite relate to these three equations:

1. (a) $4FeS_2 + 15O_2 + 14H_2O \rightarrow 4Fe(OH)_3 + 16H^+ + 8SO_4^{2-}$

   [anticipated as the dominant reaction in coals with **high** buffering capacity, propagating to equations **2** and **3**]

   Or

   (b) $2FeS_2 + 7O_2 + 2H_2O \rightarrow 2FeSO_4 + 2H_2SO_4$

   [anticipated as the dominant reaction in coals with **low** buffering capacity, propagating to Fe2+ dissolution and Fenton chemical reactions generating reactive oxygen species and thus promoting lipid peroxidation]

2. $CaCO_3 + 2H^+ \rightarrow Ca^{2+} + H_2O + CO_2$
3. $CaCO_3 + 2H^+ + SO_4^{2-} + H_2O \rightarrow CaSO_4 \cdot 2H_2O + CO_2$

As the cells were treated with particles containing varying proportions of calcite/dolomite, pyrite, and gypsum three main scenarios can be anticipated (however there may be intermediate cases). The first being particles with just pyrite and no calcite/dolomite or gypsum. This would lead to the dominance of reaction 1b and the

perpetuation of Fe dominated Fenton reactions stemming from the soluble Fe-sulfates. This scenario was confirmed by the strong positive and significant relationship between pyrite, Fe-sulfates and lipid peroxidation shown in Figure 4a -page 23.

Second, particles with a greater proportion of carbonate minerals (calcite and dolomite) compared to pyrite. Under this case it is understood that the buffering capacity of the coal particles will be higher than its acid generating capacity, as a result, equation 1a, 2 and 3 are anticipated to convert the pyrite to gypsum. This would lead to an inability of the Fe to react as the pyrite gets converted to the stable and non-reactive Fe-oxyhydroxide product. However, the formation of gypsum allows for the dissociation of Ca2+ ions which is proposed to impact cellular functions which utilise Ca via signaling mechanisms, as well as induce apoptosis (as mentioned in the manuscript – page 28 lines 1-3). The observations in the analysis supporting this observation are that (1) as the alteration process converting pyrite to Fe-oxyhydroxide limits the level of Fe available for Fenton reactions it is expected that the Ca from carbonate minerals will have a strong negative and significant effect on the extent of lipid peroxidation. This is observed in the analysis of the model coefficients (Figure 4a – page 23). Additionally, the loadings plot in Figure 3a relating the magnitude and direction of the composition-based variables, where the covariance has been maximised, shows that the effects of carbonate and Fe-sulfate and sulfide (pyrite) mineral chemistry are in opposition, while the effects of Ca derived from gypsum lie in-between these two clusters (closer to the Fe-sulfate and sulfide cluster).

The third potential scenario relates to the presence of pyrite and gypsum with no or marginal buffering capacity. This may occur in coals which have undergone some degree of weathering and have since formed pyrite through diagenetic means (processes which occur post ore formation). Here it is suggested that the extent of lipid peroxidation will be promoted through independent mechanisms, (1) relating to Fe-related Fenton chemistry sourced from pyrite and (2) Ca-related stress proposed through disruptions to calcium signalling.

Online Datasets

Dataset S1 (https://doi.org/10.25375/uct.22309492.v1). Results reporting the immunological responses of THP-1 cells exposed to coal dust in vitro.

Dataset S2 (https://doi.org/10.25375/uct.22309357.v1). Characterisation of dust-sized coal particulates: Full physicochemical, mineralogical, and textural dataset extracted from QEMSCAN analysis.

Dataset S3 (https://doi.org/10.25375/uct.22309117.v1). Characterisation of dust-sized coal particulates: Brunauer-Emmett-Teller (BET) specific surface area measurements.

Dataset S4 (https://doi.org/10.25375/uct.22309093.v1). Characterisation of dust-sized coal particulates: Malvern particle size analysis.

Dataset S5 (https://doi.org/10.25375/uct.22308925.v1). Characterisation of dust-sized coal particulates: Major element analysis using X-ray Fluorescence (XRF).

SI References

(1) David, W. I. F.; Leoni, M.; Scardi, P. Domain Size Analysis in the Rietveld Method. *Mater. Sci. Forum* **2010**, *651*, 187–200. https://doi.org/10.4028/www.scientific.net/MSF.651.187.

(2) Scrivener, K. L.; Füllmann, T.; Gallucci, E.; Walenta, G.; Bermejo, E. Quantitative Study of Portland Cement Hydration by X-Ray Diffraction/Rietveld Analysis and Independent Methods. *Cem. Concr. Res.* **2004**, *34* (9), 1541–1547. https://doi.org/10.1016/J.CEMCONRES.2004.04.014.
